# Chronic post-traumatic stress disorder-related behavioral traits develop in female rats exposed to repetitive low-level blast injury and are reversible with (2R,6R)-hydroxynorketamine

**DOI:** 10.3389/fneur.2026.1830745

**Published:** 2026-10-07

**Authors:** Georgina Perez Garcia, Gissel M. Perez, Rita De Gasperi, Miguel A. Gama Sosa, Carolyn W. Zhu, Serena Yeung, Shahrukh Khalique, Carlos A. Toro, Patrick R. Hof, Gregory A. Elder

**Affiliations:** 1 Research and Development Service, James J. Peters Department of Veterans Affairs Medical Center, Bronx, NY, United States; 2 Department of Neurology, Icahn School of Medicine at Mount Sinai, New York, NY, United States; 3 Department of Psychiatry, Icahn School of Medicine at Mount Sinai, New York, NY, United States; 4 General Medical Research Service, James J. Peters Department of Veterans Affairs Medical Center, Bronx, NY, United States; 5 Mount Sinai Alzheimer’s Disease Research Center and Ronald M. Loeb Center for Alzheimer’s Disease, Icahn School of Medicine at Mount Sinai, New York, NY, United States; 6 VA Sequencing Collaborations United for Research and Epidemiology (seqCURE), James J. Peters VA Medical Center, Bronx, NY, United States; 7 The Bronx Veterans Medical Research Foundation (BVMRF), Bronx, NY, United States; 8 Spinal Cord Damage Research Center, James J. Peters Department of Veterans Affairs Medical Center, Bronx, NY, United States; 9 Department of Medicine, Icahn School of Medicine at Mount Sinai, New York, NY, United States; 10 Nash Family Department of Neuroscience and Friedman Brain Institute, Icahn School of Medicine at Mount Sinai, New York, NY, United States; 11 Department of Geriatrics and Palliative Care, Icahn School of Medicine at Mount Sinai, New York, NY, United States; 12 Neurology Service, James J. Peters Department of Veterans Affairs Medical Center, Bronx, NY, United States

**Keywords:** animal model, behavioral testing, blast tube, metabotropic glutamate receptor 2, shock wave, traumatic brain injury

## Abstract

**Introduction:**

Many military Veterans who experienced blast-related injuries during military service suffer from chronic cognitive and mental health problems. Women constitute more than 15% of active-duty U. S. military personnel and are the fastest-growing population of U. S. Veterans. For more than a decade, we have studied a rat model of repetitive low-level blast exposure designed to mimic the type of blast-related exposure that is common in military settings. Male rats develop chronic cognitive and post-traumatic stress disorder-related traits that appear in a delayed fashion and persist for more than 1 year after exposure. These traits can be reversed with a single dose of (2R,6R)-hydroxynorketamine [(2R,6R)-HNK]. This study aimed to determine whether female rats develop a similar set of neurobehavioral alterations.

**Methods:**

Ten-week-old female rats were exposed to 75.1 kPa blast overpressures delivered once per day for three consecutive days. We tested female rats behaviorally between 1 and 4 weeks after blast exposure and again at 28 and 29 weeks. Rats were tested in an elevated zero maze, light–dark emergence task, and novel object recognition task, as well as contextual and cued fear learning and a Barnes maze. We tested how females would respond to a single dose of 20 mg/kg (2R,6R)-HNK administered intraperitoneally.

**Results:**

Following blast exposure, female rats developed chronic cognitive and behavioral traits. The full behavioral phenotype developed with a delay. Treatment with a single dose of (2R,6R)-HNK reversed behavioral changes in female rats, and beneficial effects on behavior were sustained for 4 weeks without additional drug administration. As in male rats, female rats showed elevated metabotropic glutamate receptor mGluR2. Elevations of mGluR2 were reversed by (2R,6R)-HNK treatment. Similar to male rats, the serotonin receptor 5-HT2AR was decreased in blast-injured female rats but levels were not affected by (2R,6R)-HNK treatment.

**Conclusion:**

These studies show that female rats develop a similar behavioral profile as previously observed in male rats using the same blast exposure conditions. They further support (2R,6R)-HNK as a promising therapeutic candidate for treatment of blast-induced neurobehavioral effects, even when administered after traits are well established.

## Introduction

Women now constitute 15.7% of active-duty service members and 22.9% of new recruits (1). Even before 2013, when the Pentagon allowed women to serve in combat roles, women often served alongside men as medics, military police, and in other military occupational specialties that required regular exposure to combat settings (2). Department of War (DoW) policy changes formally allowing women to serve in combat led to an increase in participation of women in combat operations, and the conflicts in Iraq and Afghanistan caused a surge in their combat exposure (2). An unprecedented number of women are now using Department of Veterans Affairs (VA) medical and social services. The number of women seeking services at the VA has more than doubled since the beginning of the conflicts in Iraq and Afghanistan, a rate of growth exceeding that of male Veterans (3).

Traumatic brain injury (TBI) is among the most frequent injuries suffered during military service. While TBIs occur in service members for various reasons, some types are relatively unique to military settings, the most prominent being blast exposure. For Veterans returning from Iraq and Afghanistan, blast exposure caused most TBIs (4). There is also concern about effects of subclinical blast exposure (5). This type of exposure, now referred to as military occupational blast exposure, is frequently encountered among service members during training and operations (5). With their increasing participation, women service members face similar risks for TBI as their male counterparts during training, combat, and other deployment-related activities. Female Veterans may also face greater risks than male Veterans for the effects of TBI because of higher rates of intimate partner violence (2). Furthermore, women often have more severe and prolonged symptoms following TBI compared to men, with female Veterans reporting more neurobehavioral symptoms, especially depression, making the study of TBI in female Veterans a high priority (6, 7).

For more than a decade, we have been studying a model of repetitive low-level blast exposure in male rats designed to mimic the type of blast-related mild TBI (mTBI) that was common in Iraq and Afghanistan (8–10). Blast-exposed male rats develop chronic cognitive and post-traumatic stress disorder (PTSD)-related traits that persist for over 1 year after exposure (8, 10), making this model relevant to the chronic neurobehavioral syndromes that occur frequently in Veterans following blast exposure (4, 11).

While there is an extensive literature on the effects of blast exposure in animals (for reviews, see (12–14)) relatively few studies have been conducted in females (15–24) and none in models with a chronic neurobehavioral phenotype like the one being studied here. In our own model, effects have only been characterized in male rats. Here we examined female rats exposed to the same repetitive low-level blast exposure that has been studied in male rats (8–10).

Ketamine is rapidly metabolized into a range of metabolites, including 12-hydroxynorketamines (HNKs) (25). (2R,6R)-hydroxynorketamine [(2R,6R)-HNK]- exerts rapid antidepressant-like actions in many preclinical models through mechanisms distinct from ketamine (26–31). Studies in metabotropic glutamate receptor mGluR2 null mutant mice suggest that (2R,6R)-HNK exerts its actions through an mGluR2 dependent mechanism (32). Because in male rats, mGluR2 was chronically elevated after blast exposure (33), we previously tested whether (2R,6R)-HNK might reverse chronic cognitive and PTSD-related traits (34). We found that a single administration of (2R,6R)-HNK reversed blast-induced behavioral traits in male rats (34).

Here we show that female rats also develop chronic cognitive and PTSD-related traits after blast injury. We in addition show that, as in male rats (34), a single dose of (2R,6R)-HNK rapidly reversed behavioral changes in females. These studies establish a blast injury model in female rats relevant to the study of the chronic neurobehavioral syndromes that are common in female military Veterans after blast injury. They further support (2R,6R)-HNK as a promising therapeutic treatment for blast-induced neurobehavioral effects in males and females.

## Methods

### Animals

A total of 32 young adult female Long Evans hooded rats (250–350 g; 10 weeks of age; Charles River Laboratories International, Wilmington, MA, United States) were used. All studies were approved by the Institutional Animal Care and Use Committee of the James J. Peters VA Medical Center. Studies were conducted in compliance with the Public Health Service policy on the humane care and use of laboratory animals, the NIH Guide for the Care and Use of Laboratory Animals, and all applicable Federal regulations governing the protection of animals in research.

### Animal housing

Animals were housed at a constant 21–22 °C with rooms on a 12:12-h light cycle with lights on at 7 a.m. All subjects were individually housed in standard clear plastic cages equipped with Bed-O’Cobs laboratory animal bedding (The Andersons, Maumee, Ohio, United States) and EnviroDri nesting paper (Sheppard Specialty Papers, Milford, NJ, United States). Food and water were available *ad libitum*. Subjects were housed on racks in random order to prevent rack position effects. Cages were coded to allow maintenance of blinding to groups during behavioral testing.

### Vaginal cytology

Vaginal smears were collected before the blast exposures on days 1, 2, and 3 after the rats were anesthetized with isoflurane but before blast exposure. Each animal was held behind the shoulder blades in a supine position. To obtain a sample, the tip of a 3-inch borosilicate glass medicine dropper was filled with approximately 0.2 mL of sterile water and inserted approximately 3.5 mm into the rat’s vagina. Vaginal manipulation was exercised with care so as not to insert the dropper too deeply, to avoid inadvertent cervix stimulation, which can initiate pseudopregnancy. The cell-containing sample was placed on an untreated glass microscope slide. Slides were stained using an adaptation of the Papanicolaou (PAP) stain used for humans, adapted for rats (35). Supplementary Table 1 describes the progressive staining steps. Samples were stained until the desired color intensity was reached. Following the final xylene change, slides were covered with mounting medium, a coverslip was placed, and slides were allowed to dry. Images were collected with a Keyence-800 microscope (Keyence Corporation of America, Itasca, IL, United States) at 100 × magnification. An observer blinded to the experimental condition scored the estrous cycle stage.

### Blast overpressure exposure

Rats were exposed to overpressure injury using a shock tube at the James J. Peters VA Medical Center (JJPVA) designed and constructed by Baker Engineering and Risk Consultants (San Antonio, TX, United States). Figure 1A shows the blast tube at the JJPVA. The instrument is approximately 6.4 m in length and comparable in design to other shock tubes in use that have been built by Baker Risk (36). The tube includes a variable-volume driver that controls the duration of the primary positive-pressure wave independently of peak overpressure. Pressurized air was used for all experiments. A dual-diaphragm spooler holding two polyethylene terephthalate Mylar TM sheets (Du Pont, Wilmington, DE, United States) was used to control pressure differences between the driver and expansion section of the tube. To induce ‘detonation’, remotely controlled electronic valves released the pressure between the spooler’s two diaphragms, causing both diaphragms to rupture simultaneously. The peak pressure at the end of the expansion chamber was determined with piezoresistive gages specifically designed for pressure–time (impulse) measurements (Model 102 M152, PCB, Piezotronics, Depew, NY, United States). All sensor data were collected and processed using Lab View2011 software (Austin, TX, United States). The end of the shock tube is fitted with an attenuator chamber that reduces ambient blast noise and suppresses reflected shock waves.

**Figure 1:**
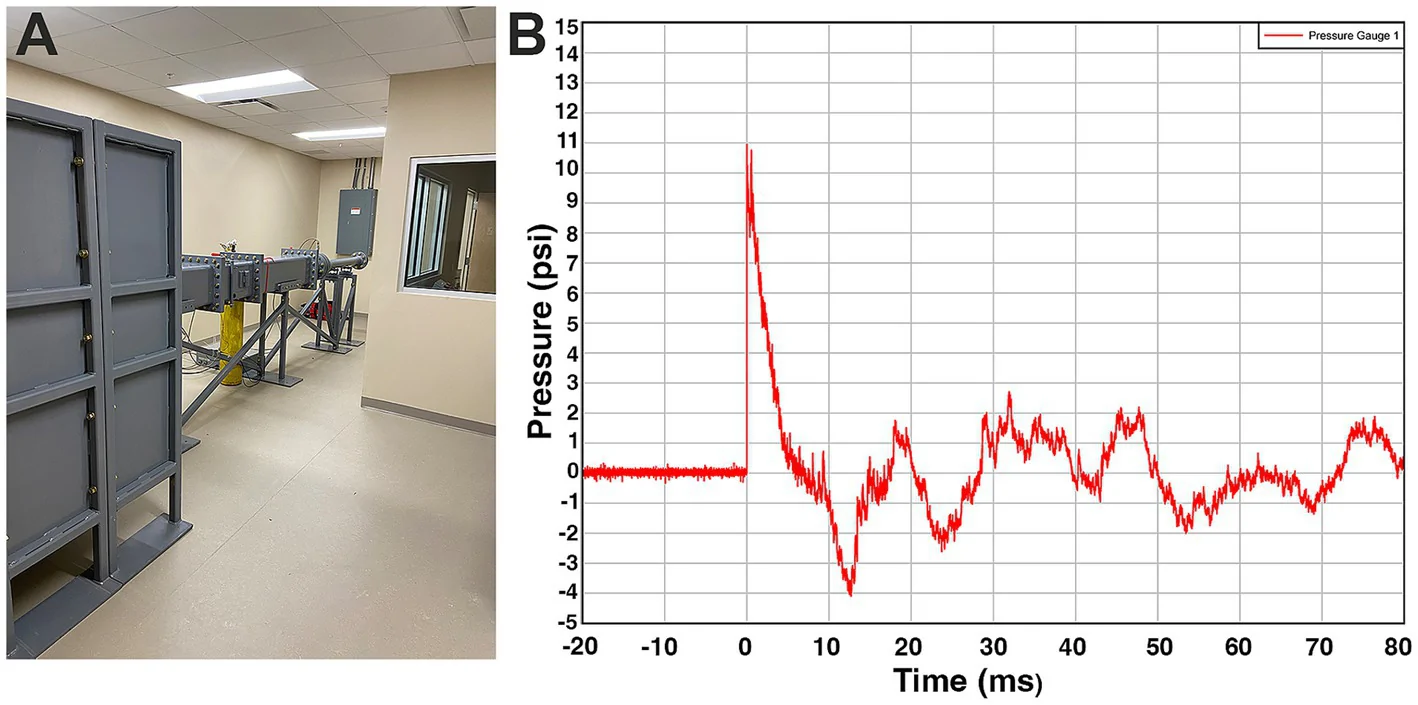
Panel **A** shows the blast tube at the James J. Peters VA Medical Center. Panel **B** shows a typical time pressure tracing. The wave exhibits a maximum overpressure of 75.1 kPa (10.9 psi). The initial overpressure wave lasts 7.5 ms and has an impulse of 26.4 psi*ms.

Individual rats were anesthetized using an isoflurane gas anesthesia system (Somni Scientific, MacMurray, PA, United States) consisting of a vaporizer, gas lines and valves, and an activated charcoal-scavenging system adapted for use with rodents. Rats were placed into a polycarbonate induction chamber, which was closed and immediately flushed with a 5% isoflurane mixture in air for 2 min. Rats were placed into a cone-shaped plastic restraint device and mounted on a square grid. Head and body movement were restricted by harnesses that affixed the animal to the grid and restricted movement during the blast overpressure exposure without restricting breathing. Rats were randomly assigned to sham or blast conditions and placed in the shock tube lying prone, with the plane representing a line from the tail to the nose in line with the longitudinal axis of the shock tube and the head placed facing the shock wave. The total length of time under anesthesia, including placement in the shock tube and execution of the blast procedure, was typically less than 3 min. Figure 1B shows a typical time vs. pressure tracing. This wave closely mimics that of our previous studies using a shock tube at the Naval Medical Research Command (Silver Spring, MD, United States) (37) with a maximum overpressure of 75.1 kPa (10.9 psi), a 7.5 ms duration initial overpressure wave, and an impulse of 26.4 psi*msec. Exposures were administered one exposure per day for 3 consecutive days.

### Behavioral testing

The timing of testing in relationship to blast exposure is described in Figure 2 and Table 1.

**Figure 2:**
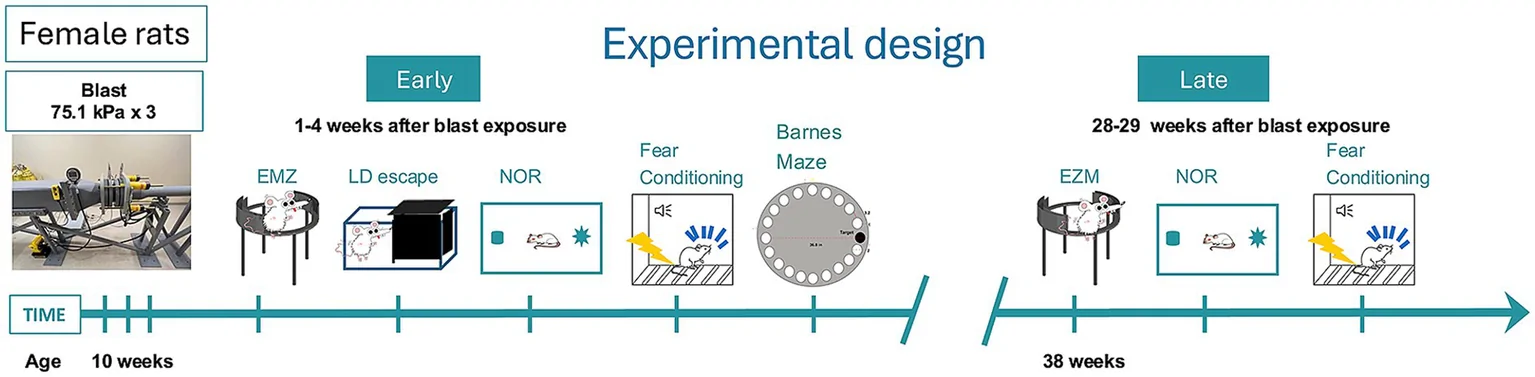
Experimental design. The time course of the study is outlined. Table 1 shows the exact timing of testing and summarizes the results.

**Table 1:** Summary of timing of behavioral testing and behavioral phenotypes observed after repetitive low-level blast exposure in female rats.

Behavioral test	Weeks after blast exposure	Phenotype (Yes/No)	Notes
Early			
EZM	1	No	
LD	1	No	
NOR	1	Yes	Impaired STM and LTM
Fear contextual	2	No	
Fear cued	2	Yes	Exaggerated cued freezing
Barnes maze	4	No	
Late			
EZM	28	Yes	Anxiety phenotype
LD	Not tested		
NOR	28	Yes	Impaired STM and LTM
Fear contextual	29	No	
Fear cued	29	Yes	Exaggerated cued freezing

EMZ, elevated zero maze; LD, light dark escape; LTM, long term memory; NOR, novel object recognition; STM, short term memory.

#### Elevated zero maze (EZM)

The apparatus consisted of a circular black Plexiglas runway 121.92 cm in diameter, raised 76 cm off the floor (San Diego Instruments, San Diego, CA, United States). The textured runway was 5.08 cm across and divided equally into alternating quadrants of open runway enclosed only by a 1.27 cm lip and closed runway with smooth 15.24 cm walls. All subjects received a 5-min trial beginning in a closed arc of the runway. During each trial, subjects were allowed to move freely around the runway, with all movement tracked automatically by a video camera placed on the ceiling directly above the maze. Data were analyzed by ANY-maze (San Diego Instruments, San Diego, CA, United States), yielding measures of total movement time and distance for the entire maze, as well as time spent and distance traveled in each of the individual quadrants. From the quadrant data, measures of total open and closed arc times, latency to enter an open arc, total open arm entries, and latency to completely cross an open arc between two closed arcs were calculated. Subject position was determined by centroid location.

#### Light/dark emergence

A light/dark emergence task was run in Versamax activity cages with opaque black Plexiglas boxes enclosing the left half of the interiors so that only the right sides were illuminated. Animals began on the dark side and were allowed to explore freely for 10 min with access to the left (light) side through an open doorway located in the center of the monitor. Subject side preference and emergence latencies were tracked by centroid location, with all movement automatically tracked and quantified. Light-side emergence latency, time to reach the center of the lighted side (light-side center latency), and percent total light-side duration were calculated from beam breaks. All equipment was wiped clean between subjects.

#### Novel object recognition

Rats were habituated to the arena (90 cm length x 60 cm width x 40 cm height) for 20 min, 24 h before training. On the training day, two identical objects were placed on opposite ends of the empty arena, and the rat was allowed to explore the objects freely for 5 min. After a 1-h delay, during which the rat was held in its home cage, one of the two familiar objects was replaced with a novel one, and the rat was allowed to freely explore the familiar and novel objects for 5 min to assess short-term memory (STM). After a 24-h delay, during which the rat was held in its home cage, one of the two familiar objects was replaced with a novel one different from the ones used during the STM. The rat was allowed to freely explore the familiar and novel object for 5 min to assess long-term memory (LTM). Raw exploration times for each object were expressed in seconds. Object exploration was defined as sniffing or touching the object with the vibrissae, or as the animal’s head being oriented toward the object with the nose placed less than 2 cm from the object. All sessions were recorded by video camera (Sentech, Carrollton, TX, United States) and analyzed with ANY-maze software (San Diego Instruments). In addition, offline analysis by an investigator blind to the blast-exposed status of the animals was performed. Objects to be discriminated were of different size, shape, and color and were made of plastic or metal material. The objects consisted of a 330 mL soda can, a metal box, a cup, and a plastic tube. All objects were cleaned with 70% ethanol between trials. A discrimination index (DI) was calculated with the formula: (time exploring the NO minus time exploring the FO)/(total exploration time) X 100.

#### Barnes maze

The test was performed using a standard apparatus made of opaque gray Plexiglas (Maze Engineers, Boston, MA, United States). The rat version uses 20 holes (10 cm hole diameter) in a circular table with 122 cm diameter and 65 cm stand height. The test was conducted in two phases: training (days 1 to 4) and testing (day 5). Before starting each session, subjects in their cages were acclimated to the testing room for 1 h. Rats were transported from their cage to the center of the platform within a closed starting chamber where they remained for 10 s before being allowed to explore the maze. The escape box was always located under the same hole. Extra maze signals were posted on the walls for animal reference. Subjects failing to enter the escape box within 4 min on trials 1–4 were guided to the escape box by the experimenter, and the latency was recorded as 240 s. Trial 5 was treated as a probe (test) trial, and subjects were given up to 180 s to enter the escape box. The platform and the escape box were wiped with 70% ethanol after each trial. Trials were recorded by video camera and analyzed with ANY-maze software.

#### Contextual and cued fear conditioning

Sound-attenuated isolation cubicles (Ugo Basile, Gemonio, Italy) were utilized. Each Isolation cubicle [635(w) x 550(d) x 580(h)mm] included a fan, lights, and an overhead camera (infrared CCD, Noldus Leesburg, VA, United States). Each cubicle was equipped with a rat cage for fear conditioning [size 270 (w) x 320 (l) x 410 (h) mm], with an electrified grid floor and transparent walls (Ugo Basile catalog number 46002-D03). All aspects of the test were monitored by a video-tracking system (Ethovision, Leesburg, VA, United States). Videos were recorded at 25 frames per second at 1920 × 1,080 resolution. During training, the chambers were scented with almond extract, lined with white paper towels, had background noise generated by a small fan, and were cleaned before and between trials with 70% ethanol. Each subject was placed inside the conditioning chamber for 2 min before the onset of a conditioned stimulus (CS; an 80 dB, 2 kHz tone), which lasted for 20 s with a co-terminating 2-s footshock [0.75 mA; unconditioned stimulus (US)]. A total of three tone/shock pairings were administered, with the first and second/third separated by 1 min. Each rat remained in the chamber for an additional 60 s following the third CS-US pairing before being returned to its home cage. Animals remained in their home cages for 24 h, at which time the contextual fear response was tested by measuring freezing behavior during a 4 min test in the conditioning chamber under conditions identical to those of the training session with the exception that no footshock or tone (CS or US) was presented. Animals were returned to their home cage for another 24 h, at which time cued conditioning was tested. To create a new context with different properties, the chambers were free of background noise (fan off), lined with blue paper towels, scented with lemon extract, and cleaned with isopropanol before and during all trials. Each subject was placed in this novel context for 2 min and baseline freezing was measured, followed by exposure to the CS (20-s tone) at 120, 260, and 420 s. Analyses were performed frame by frame using Ethovision software, which distinguishes between active and immobile states to automatically score freezing. Freezing was defined as the absence of movement (except for respiration) during each 10-s interval. Minutes 0–2 of each session were used to measure baseline freezing, which was presented as a percentage in all experiments.

### Drug administration

(2R,6R)-Hydroxynorketamine hydrochloride ((2R,6R)-HNK, Sigma-Aldrich, St. Louis, MO, United States) was dissolved in saline. Blast-exposed female rats were treated with a single dose of 20 mg/kg (2R,6R)-HNK administered intraperitoneally. Vehicle-treated animals were administered saline. Animals were divided into three experimental groups: (1) sham-exposed rats treated with vehicle (sham + veh), (2) Blast-exposed rats treated with vehicle (blast + veh), and (3) blast-exposed rats treated with (2R,6R)-HNK (blast + HNK). The timing of drug administration in relation to blast exposure and behavioral testing is shown in Figure 3 and Table 2. The dose was chosen based on previous studies in male rats (34).

**Figure 3:**
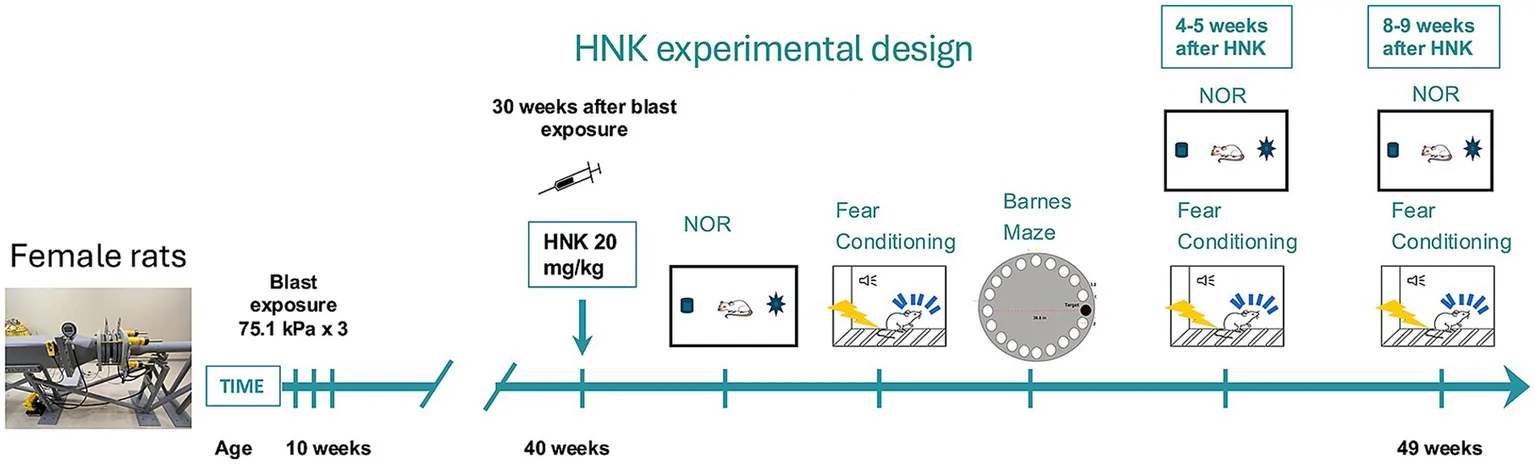
Experimental design for (2R,6R)-hydroxynorketamine treatment study. The female rats tested earlier were divided into three groups consisting of: (1) sham-exposed rats treated with saline (*n* = 15), (2) blast-exposed rats treated with saline (*n* = 7), and (3) blast-exposed rats treated with a single dose of 20 mg/kg (2R,6R)-HNK (*n* = 8). Table 2 summarizes the timing of (2R,6R)-HNK administration in relation to blast exposure, the tests performed, and the results.

**Table 2:** Summary of timing of behavioral testing and effects of (2R,6R)-hydroxynorketamine treatment in blast-exposed female rats.

Behavioral test	Weeks after blast exposure	Time after HNK administration	Blast related phenotype present (Yes/No)	HNK effect
Novel object recognition (training and LTM)	30	24–48 h	Yes	LTM deficits reverted
Fear Conditioning (training and cued)	31	1 week	Yes	Increased cued freezing reverted
Barnes Maze	32	2 weeks	No	None
Novel object recognition (LTM)	34	4 weeks	Yes	LTM deficits reverted
Fear Conditioning (cued)	35	5 weeks	Yes	Increased cued freezing reverted
Novel object recognition (LTM)	38	8 weeks	Yes	HNK effect gone
Fear Conditioning (cued)	39	9 weeks	No	HNK effect indeterminate

HNK, hydroxynorketamine; LTM, long term memory.

### Tissue collection

For biochemical studies, rats were euthanized by CO_2_ inhalation; the brain was removed and regionally dissected as previously described (8). The portion of brain collected as anterior cortex included all neocortex anterior to the optic chiasm, including prelimbic (PrL), infralimbic (IL), cingulate (Cg1) motor, somatosensory, and insular cortices.

### Western blot analysis

Tissues were homogenized in 0.1 M Tris HCl pH 7.6 containing 150 mM NaCl, 1% Triton, 0.1% protease, and phosphatase inhibitors (Halt, Protease and phosphatase inhibitor cocktail, ThermoFisher, Waltham, MA, United States) using Zirconia beads and the FastPrep tissue homogenizer (MP Biomedicals, Irvine, CA, United States). The lysates were centrifuged at 14,000 rpm for 20 min, and the supernatant was saved and frozen at -80 °C. Protein concentration was measured with the BCA assay (ThermoFisher). Protein (50 µg) was separated on SDS-PAGE gels, blotted onto Immobilon P membranes (Millipore, Burlington, MA, United States), and blocked in 5% non-fat dry milk in 20 mM Tris–HCl, pH 7.5, 0.25 M NaCl (TBST). The membrane was incubated overnight at 4 °C with the primary antibody diluted in blocking solution, washed in TBST, and incubated at room temperature for 1.5 h with peroxidase-conjugated anti-rabbit IgG (NA934) or anti-mouse IgG (NA 931) (Cytiva, Marlborough, MA, United States, 1:7500) diluted in blocking solution. The following primary antibodies were used: a rabbit polyclonal anti-metabotropic glutamate receptor (mGluR) 2/3 (Ab 1553, Millipore Sigma, 1:800); a rabbit polyclonal anti-mGluR2 (07261-I, Millipore Sigma, 1:1000); a rabbit monoclonal anti-mGluR3 (ab-166608, Abcam, Waltham MA, United States, 1:1300); a mouse monoclonal anti-5-HT2AR (SR-2A antibody, sc-166775, Santa Cruz Biotechnology, Dallas TX USA, 1:600) and a rabbit monoclonal anti-GAPDH (clone D16H11 #5174, Cell Signaling Technology, Danvers, MA, United States 1:2500). The membrane was developed with ECL Prime western blotting detection reagent (Cytiva) and imaged with Amesham ImageQuant 800 imaging system. Membranes were stained with Ponceau Red S Stain (Millipore Sigma) to image total protein loading. Bands were quantitated with ImageQuant TL (Cytiva). Levels were expressed as a ratio to glyceraldehyde 3-phosphate dehydrogenase (GAPDH) or to total protein loading derived from the integrated intensity of the Ponceau S-stained bands. Stripping of blots was performed with Reblot Plus buffer (ThermoFisher) as recommended by the manufacturer.

### Statistical analysis

Data sets were examined for assumptions of parametric tests (normality, homogeneity of variance). Outliers were first excluded at the stage of data collection in any circumstances where data were not considered a reliable measurement (e.g., equipment lost tracking of the animal). At the statistical level, outliers were identified using the ROUT method with Q set to 1%. Comparisons were performed using univariate ANOVA, repeated-measures ANOVA, or unpaired *t*-tests. When comparisons involved three groups, if a significant ANOVA was present, group differences were determined using Fisher’s LSD (38). When repeated-measures ANOVA was calculated, the Greenhouse–Geisser correction (39) was utilized. Effect sizes were calculated using Hedges *g* (40) and R^2^ (eta squared). Contingency tables comparing relative frequencies were analyzed using Fisher’s exact test. Values are expressed as mean ± SEM. Statistical tests were performed using the program GraphPad Prism 11.0 (GraphPad Software, San Diego, CA, United States) or SPSS v30 (IBM, Armonk, NY, United States).

## Results

### General effects of blast exposure on animal health

No effect of blast exposure was noted on general health or behaviors such as grooming. Weights at the end of late behavioral testing are shown in Supplementary Figure 1A. Blast-exposed rats weighed ≈ 17% more at the end of late behavioral testing 30 weeks after blast exposure.

### The estrous cycle in female rats before and during the blast exposure sequence

The estrous cycle is an important variable in studies with female rats. We recorded the phase of the estrous cycle on days 1, 2, and 3 of the blast exposures using vaginal smears collected after the rats were anesthetized with isoflurane but before the blast exposures were administered (Supplementary Figure 2). On day 1 (before blast exposure in either group), the relative frequencies of estrous cycle stages did not differ between groups (*p* = 0.9931, Fisher’s exact test). Relative frequencies also did not differ on day 2 (*p* = 0.4933) or day 3 (*p* = 0.4811) prior to that day’s blast exposure.

### Early behavioral testing (1–4 weeks)

The timeline for testing is shown in Figure 2. Table 1 shows the timing of each test in relation to the last blast exposure and summarizes results. Female rats showed no abnormalities in EZM (Supplementary Figure 3) or light–dark emergence (Supplementary Figure 4) when tested within 1 week of blast exposure. However, blast-exposed female rats showed NOR deficits when tested within 1 week. While blast-exposed female rats explored the objects equally in the training session (Figure 4A), they spent no more time exploring the novel than the familiar object in either the STM or LTM sessions, while the sham-exposed explored the novel objects more (Figures 4B,C; effect sizes are shown in Supplementary Table 2.). The lack of preference for the novel object was reflected in a lower discrimination index for blast-exposed female rats in the LTM [*p* = 0.0498, *g* = 0.-7.065, 95% CI (−0.4651 to 0.001394)] testing (Figure 4D).

**Figure 4:**
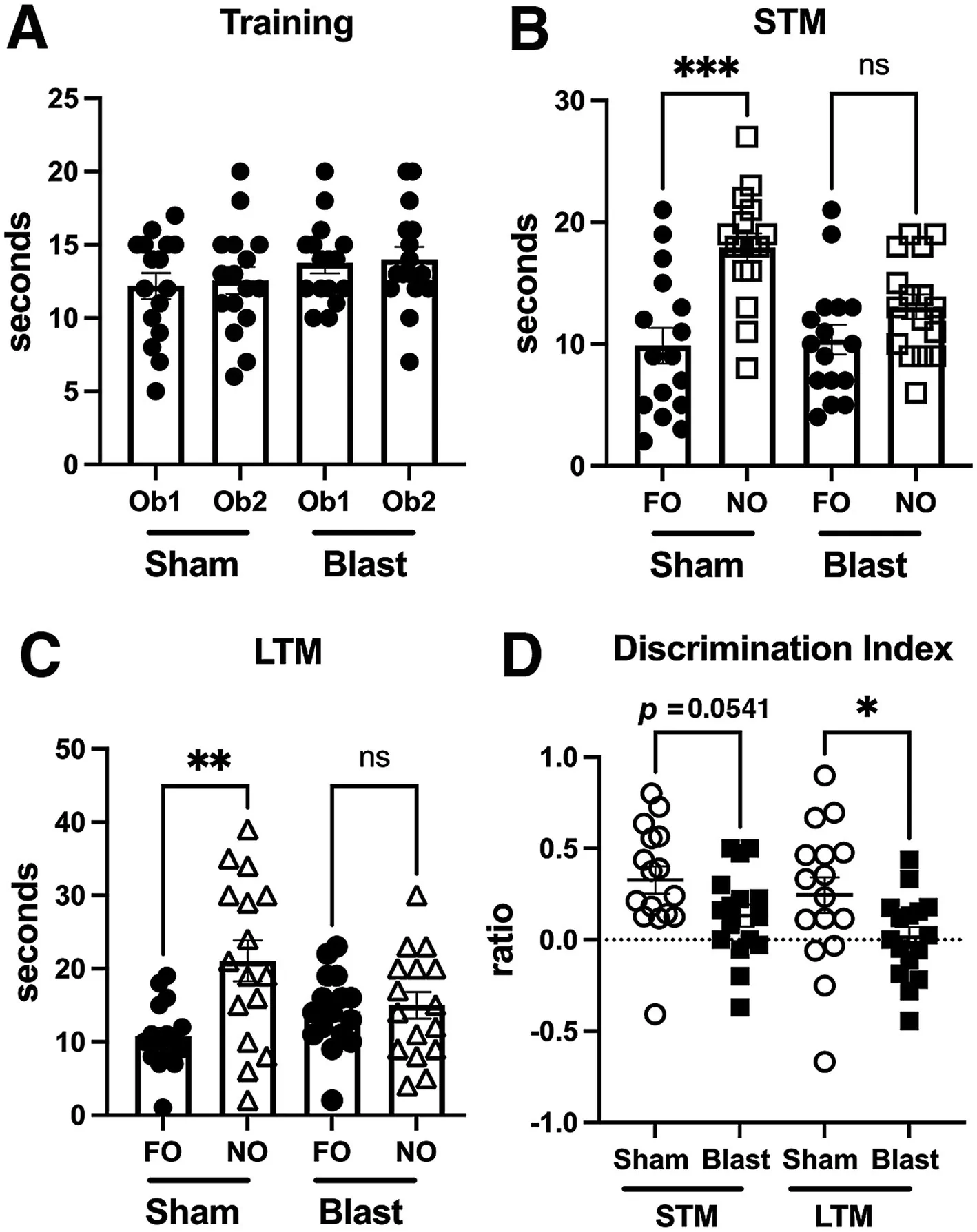
Testing of blast-exposed females in a novel object recognition (NOR) task within 1 week after blast exposure. Blast-exposed (*n* = 16) and sham-exposed (*n* = 16) rats were tested in an NOR task within the first week after blast exposure. Panel **A** shows time spent exploring the identical objects (Ob1 and Ob2) during the training session. Panels **B** and **C** show exploration of the previously presented familiar object (FO) compared to the novel object (NO) when presented 1 h (B, short-term memory, STM) or 24 h (C, long-term memory, LTM) later. Panel **D** shows a discrimination index calculated for the STM and LTM testing. Asterisks indicate values significantly different from controls (* *p* < 0.05, ** *p* < 0.01, *** *p* < 0.001, unpaired *t*-tests, ns not significant). Error bars in all panels indicate the standard error of the mean (SEM).

In fear learning tested 2 weeks after blast exposure, sham and blast-exposed female rats responded similarly in the training phase, with increased freezing following pairing of the tone with the shock (Figure 5A). In the contextual testing (Figure 5B) repeated-measure ANOVA did not show any overall group effect, with both groups responding with similar freezing during testing in the same environment as the training. However, in cued testing, when the tone was presented without a shock in a novel environment, blast-exposed female rats exhibited increased freezing, suggesting that exaggerated fear learning was present (Figure 5C).

**Figure 5:**
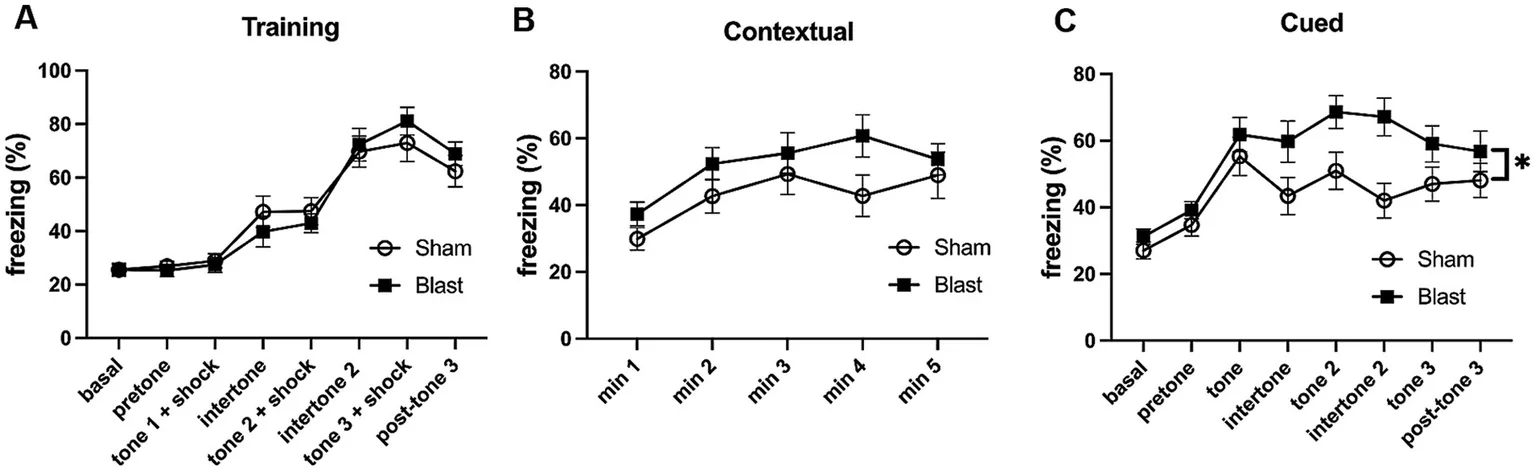
Testing of blast-exposed females in a fear conditioning paradigm within 2 weeks after blast exposure. Blast-exposed (n = 16) and sham-exposed (n = 16) rats were tested in contextual and cued fear conditioning within 2 weeks after blast exposure. Results are shown for the training phase **(A)**, contextual fear memory **(B)**, tested 24 h after training, and cued fear **(C)** memory, tested another 24 h later. A repeated measures ANOVA of freezing during the training sessions revealed a significant within-subjects effect of freezing for baseline vs. tone (F _3.370, 101.099_ = 54.300, *p* < 0.001, df Greenhouse–Geisser correction) but no effect of freezing*condition (F _3.370, 101.099_ = 0.858, *p* = 0.476). A test of between-subject effects revealed no significant between-group differences during the training session (F _1, 30_ = 0.007, *p* = 0.934). In the contextual testing **(B)** a repeated measure ANOVA did not show any overall group effect with both groups responding with increased freezing during the testing (F _3.311, 96.031_ = 9.562, *p* < 0.001), no interaction effect (F _3.311, 96.031_ = 1.029, *p* = 0.388) and no between-group differences (F _1, 29_ = 2.267, *p* = 0.143). In the cued testing **(C)**, both groups responded with freezing to the presentation of the tones (F _5.091, 147.637_ = 18.112, *p* < 0.001) but with no interaction effect (F _5.091, 147.637_ = 2.126, *p* = 0.064). However, there were significant overall between-group effects, with the blast-exposed group freezing more (F _1, 29_ = 5.344, *p* = 0.028). Error bars in all panels indicate the standard error of the mean (SEM).

At 4 weeks after blast exposure, when tested in a Barnes maze, blast-exposed female rats showed a training curve similar to sham-exposed female rats (Supplementary Figure 5A). In the probe trial, the latency to find the target did not differ between groups (Supplementary Figure 5B).

### Late behavioral testing (28–29 weeks)

In late testing (Table 1), which began at 28 weeks after blast exposure, blast-exposed female rats were initially tested on 2 consecutive days in an EZM (Figure 6). On both testing days, blast-exposed females traveled similar distances, at similar speeds, and spent the same amount of time in motion as shams. However, compared to shams, blast-exposed females exhibited a longer latency to enter an open arm (*p* = 0.0202 on day 1; *p* = 0.0017 on day 2), made fewer open arm entries (*p* = 0.0412 on day 1; *p* = 0.0017 on day 2) as well as spent less time in the open arms (*p* = 0.0412 on day 1; *p* = 0.0034 on day 2) and took longer to enter a second open arm (the cross-arm latency, *p* = 0.0178 on day 1; *p* = 0.0122 on day 2). Effect sizes are shown in Supplementary Table 3. On both testing days, whenever *p*-values were < 0.05, Hedges’ *g*-values ranged from 0.77 to 1.2, indicating a large effect size. Thus, when tested late, blast-exposed females showed a component of anxiety which was not present earlier.

**Figure 6:**
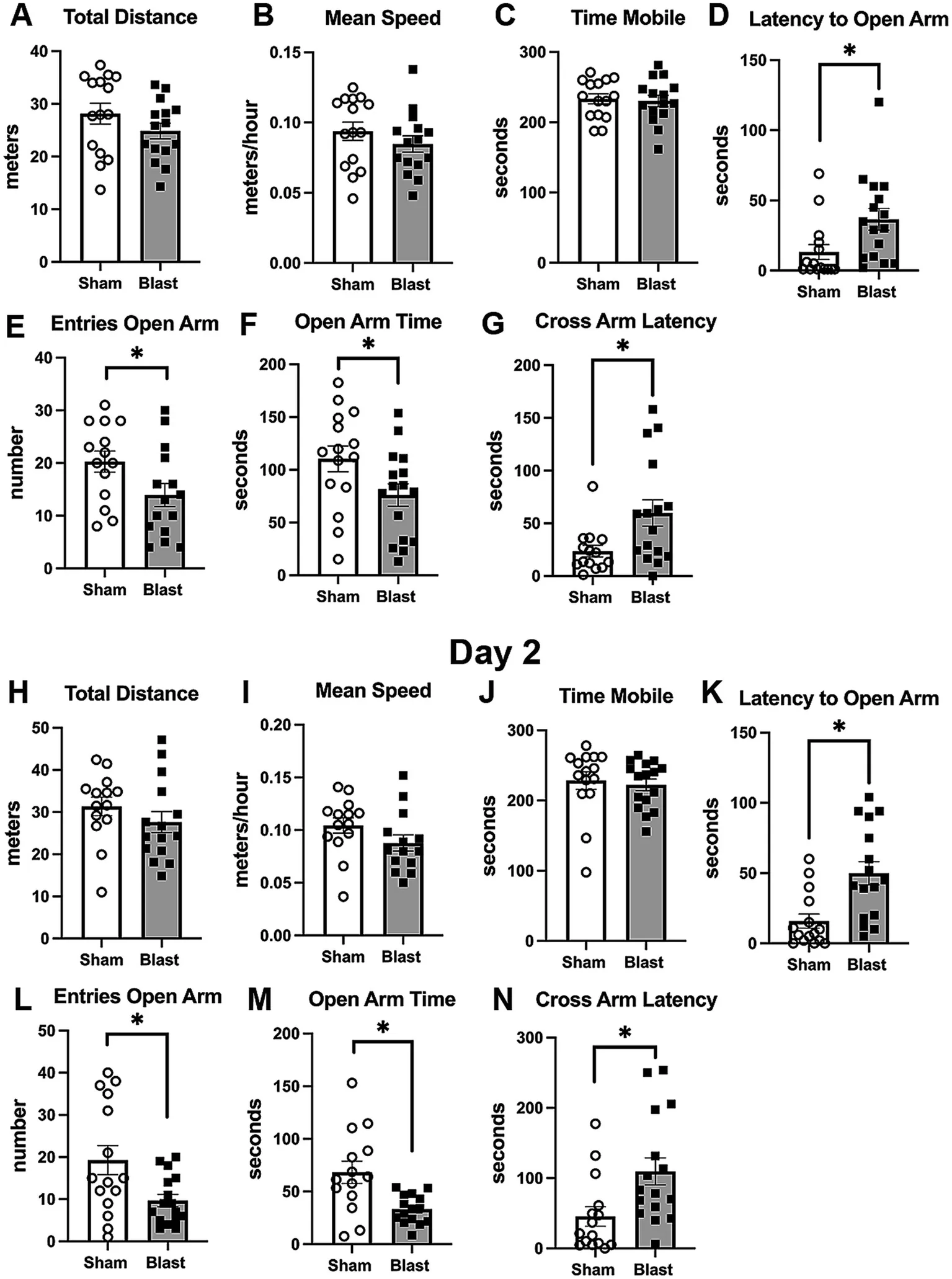
Elevated zero maze (EZM) testing of female rats at 28 weeks after blast exposure. Blast-exposed (*n* = 15) and control (*n* = 15) female rats were tested in an EZM on 2 consecutive days. The plots show the distance traveled **(A,H)**, mean speed **(B,I)**, time in motion **(C,J)**, latency to enter an open arm **(D,K)**, open arm entries **(E,L)**, time spent in the open arms **(F,M)**, and the latency to cross into a second open arm **(G,N)** for days 1 and 2 (* *p* < 0.05, unpaired *t*-tests). Error bars in all panels indicate the standard error of the mean (SEM).

In NOR testing late (28 weeks; Figure 7), results were very similar to early testing (Figure 4). Blast-exposed subjects explored the objects like the sham during training (Figure 7A). However, blast-exposed female rats exhibited deficits in both STM and LTM (Figures 7B,C) reflected in an altered discrimination index (Figure 7D). Effect sizes (Supplementary Table 4) showed that whenever *p*-values were < 0.05, Hedges’ *g*-values ranged from 1.0 to 1.5, indicating a large effect size.

**Figure 7:**
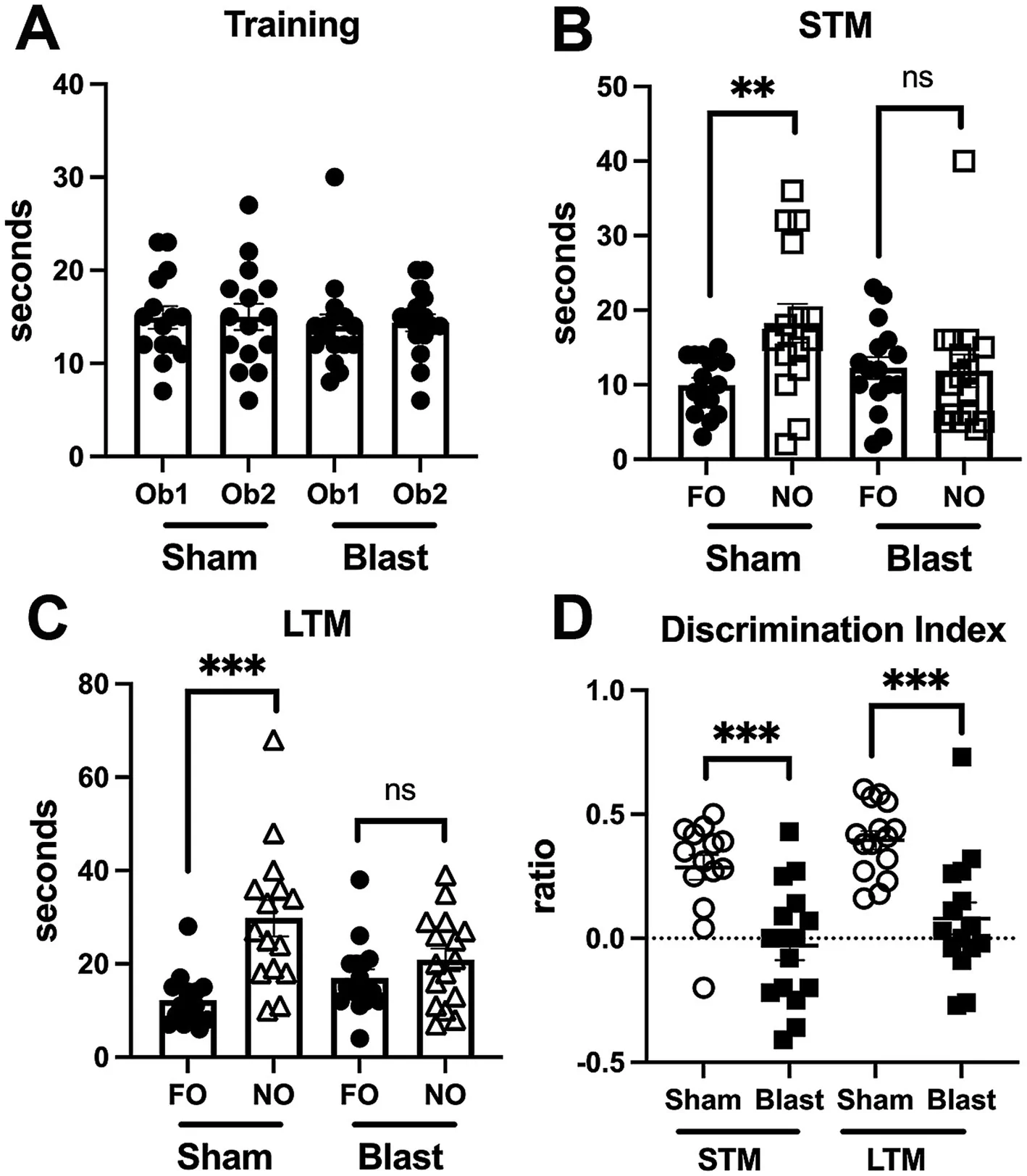
Testing of blast-exposed females in the novel object recognition (NOR) task at 28 weeks after blast exposure. Blast-exposed (*n* = 16) and sham-exposed (*n* = 15) rats were tested in an NOR task 28 weeks after blast exposure. Panel **A** shows time spent exploring the objects (OB1 and OB2) during the training session. Panels **B** and **C** show exploration of the previously presented familiar object (FO) compared to the novel object (NO) when presented 1 h (**B**, short-term memory, STM) or 24 h (**C**, long-term memory, LTM) later. Panel **D** shows a discrimination index calculated for the STM and LTM testing. Asterisks indicate values significantly different from controls (** *p* < 0.01, *** *p* < 0.001, unpaired *t*-tests, ns not significant). Error bars in all panels indicate the standard error of the mean (SEM).

In fear learning tested late (29 weeks) after blast exposure, results were very similar to early testing (Figure 5). Sham- and blast-exposed female rats responded similarly during training (Figure 8A). There were no differences between blast-exposed and sham in the contextual testing (Figure 8B). In cued testing, as in the early testing, blast-exposed female rats exhibited increased freezing, suggesting that exaggerated fear learning was present (Figure 8C).

**Figure 8:**
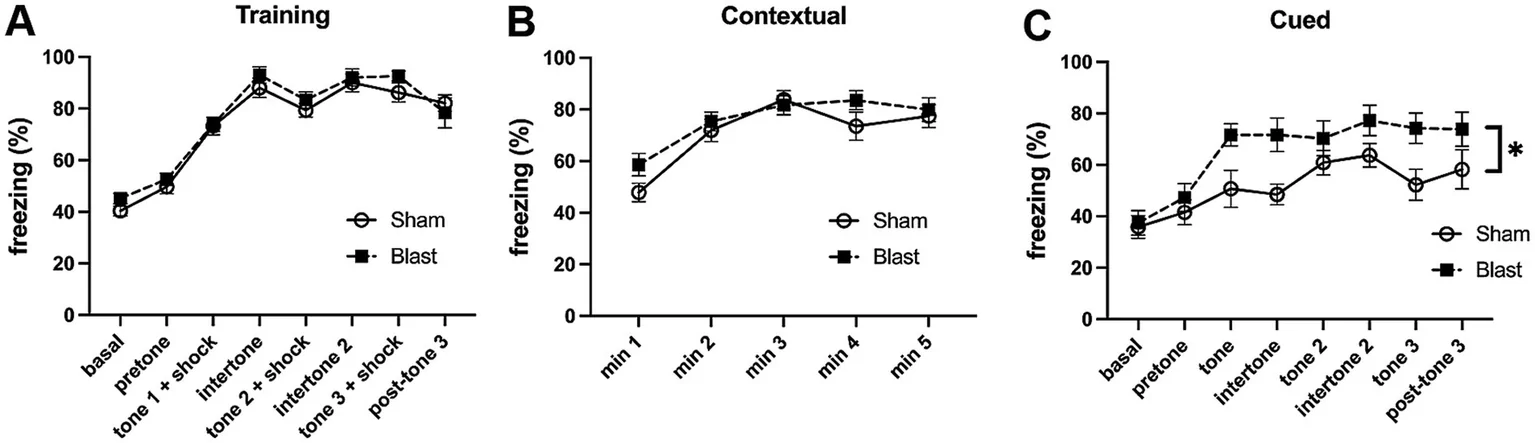
Testing of blast-exposed females in a fear conditioning paradigm at 29 weeks after exposure. Blast-exposed (*n* = 16) and sham-exposed (*n* = 15) rats were tested in contextual and cued fear conditioning at 29 weeks after blast exposure. Results are shown for the training phase **(A)**, contextual fear memory **(B)**, tested 24 h after training, and cued fear memory **(C)**, tested another 24 h later. A repeated measures ANOVA of freezing during the training sessions revealed a significant within-subjects effect of freezing for baseline vs. tone (F _4.272, 123.879_ = 76.946, *p* < 0.001) but no effect of freezing*condition (F _4.272, 123.879_ = 0.574, *p* = 0.693). A test of between-subject effects revealed no significant between-group differences during the training session (F _1, 29_ = 1.483, *p* = 0.233). In the contextual testing **(B)** a repeated measure ANOVA did not show any overall group effect with both groups responding with increased freezing during the testing (F _2.949, 82.569_ = 23.835, *p* < 0.001) but no interaction effect (F _2.949, 82.569_ = 1.267, *p* = 0.290) and no between-group differences (F _1, 28_ = 1.484, *p* = 0.233). In the cued testing **(C)**, both groups responded with freezing to the presentation of the tones (F _3.934, 102.292_ = 15.174, *p* < 0.001) but with no interaction effect (F _3.934, 102.292_ = 1.754, *p* = 0.145). However, there were significant overall between-group differences, with the blast-exposed group freezing more (F _1, 26_ = 5.676, *p* = 0.025). Error bars in all panels indicate the standard error of the mean (SEM).

### (2R,6R)-hydroxynorketamine [(2R,6R)-HNK] reverses blast-induced cognitive traits and altered fear learning in female rats

Previously, we found that a single dose of (2R,6R)-HNK reversed neurobehavioral traits in male rats exposed to repetitive low-level blast (34). We tested whether females might respond similarly to a single dose of 20 mg/kg (2R,6R)-HNK administered intraperitoneally (IP). The female rats tested above were divided into three groups consisting of: (1) sham-exposed rats treated with saline, (2) blast-exposed rats treated with saline, and (3) blast-exposed rats treated with 20 mg/kg (2R,6R)-HNK. Comparing sham + saline-treated controls with blast + saline-treated animals provided a positive control for the presence of the blast-related behavioral phenotype. Comparing blast + saline to blast + HNK allowed effects of (2R,6R)-HNK on the blast-related phenotype to be determined. Rats were tested in NOR and cued fear learning tasks found previously to be informative. Figure 3 shows the study design, and Table 2 shows the timing of each test in relation to the last blast exposure, as well as the results of (2R,6R)-HNK administration. Treatment with (2R,6R)-HNK resulted in no obvious effects on general health or behaviors such as grooming. Weights at 4 weeks after treatment are shown in Supplementary Figure 1B. Whether treated with (2R,6R)-HNK or saline, blast-exposed rats weighed ≈ 12–20% more than shams.

NOR testing was begun 24 h after drug administration. Rats were retrained on day 1 with two identical objects. Twenty-four hours later, one of the familiar objects was replaced with a NO, and the subjects were tested for recognition memory. Results are presented in Figure 9 and Supplementary Table 5. During training, all groups explored the two objects equally (Figure 9A). When presented with the choice between an NO and FO, 24 h later (Figure 9B), sham + saline explored the NO more than the FO [*p* = 0.0022, *g* = 1.245, 95% CI (3.013 to 12.27)], while blast + saline explored the NO and FO equally [*p* = 0.6778, *g* = 0.2130, 95% CI (−4.188 to 6.118)]. However, blast + HNK explored the NO more than the FO [*p* = 0.0119, *g* = 0.9259, 95% CI (−0.8082 to 17.81)], indicating that (2R,6R)-HNK rescued NOR deficits in blast-exposed female rats.

**Figure 9:**
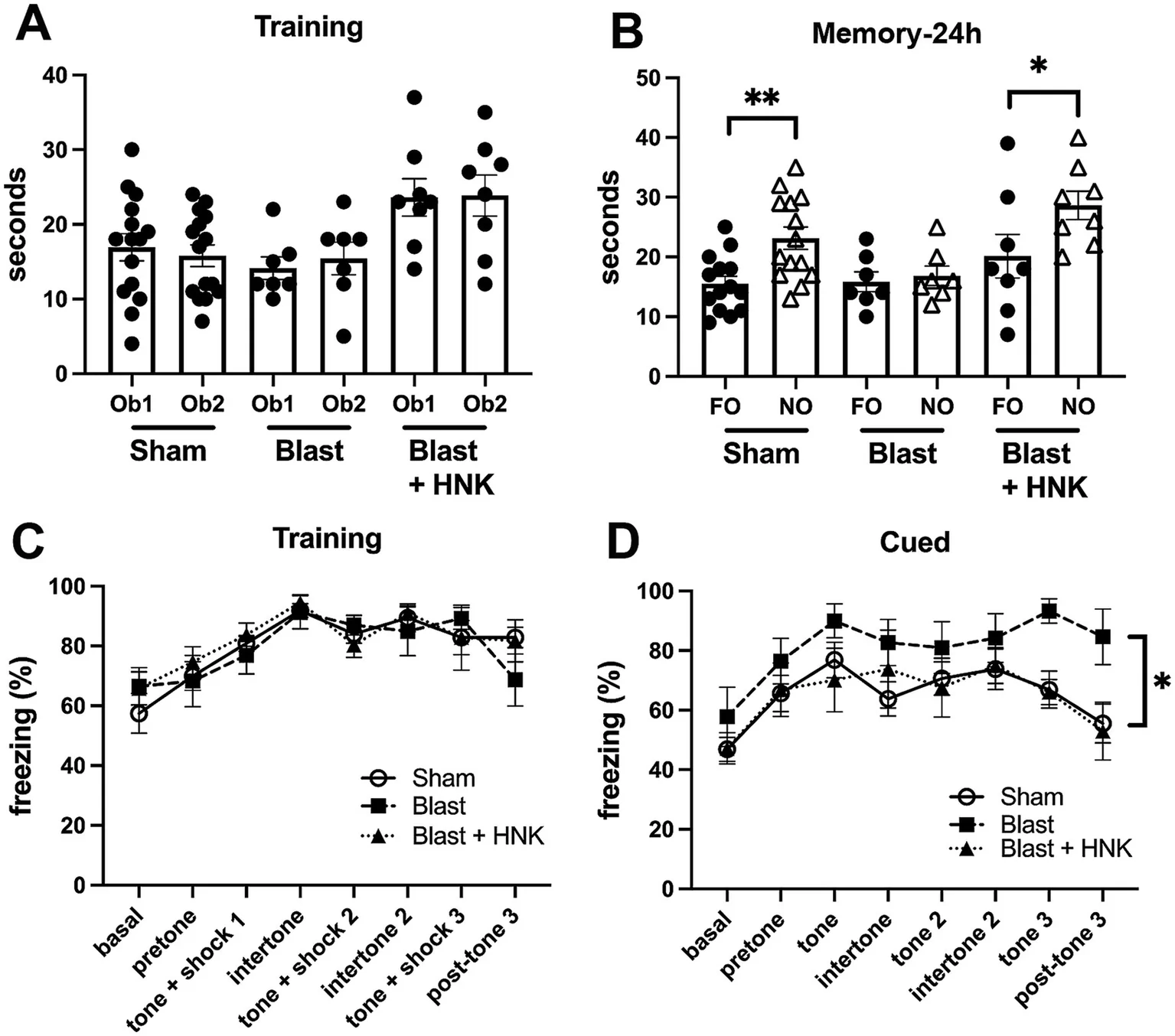
Testing of female rats treated with (2R,6R)-hydroxynorketamine in novel object recognition (NOR) and cued fear learning. Sham- and blast-exposed female rats were treated with saline (*n* = 15 sham, *n* = 7 blast), and blast-exposed rats were treated with (2R,6R)-HNK (*n* = 8). Rats were then retrained and tested in NOR and a fear conditioning paradigm. Figure 3 shows the study time course. Panel **A** shows time spent exploring the objects (Ob1 and Ob2) during the NOR training session. **B** shows exploration 24 h later of the previously presented familiar object (FO) compared to a novel object (NO) which replaced one of the familiar objects used during training. Asterisks indicate values significantly different from controls (* *p* < 0.05, ** *p* < 0.01, unpaired *t*-tests). In panels **C** and **D**, results of fear learning and testing are shown for the training phase **(C)** and cued fear memory **(D)**, which was tested 24 h after training. A repeated measures ANOVA of freezing during the training sessions revealed a significant within-subjects effect of freezing for baseline vs. tone (F _3.387, 91.439_ = 9.629, *p* < 0.001) but no effect of freezing*condition (F _6.773, 91.439_ = 0.645, *p* = 0.712). A test of between-subject effects revealed no significant between-group differences during the training sessions (F _2, 27_ = 0.238, *p* = 0.790). In the cued testing **(D)**, all groups responded with freezing to presentation of the tones (F _4.370, 109.256_ = 7.486, *p* < 0.001) but without any interaction effect (F _8.740, 109.256_ = 0.735, *p* = 0.672). However, there were significant overall between-group effects when comparing freezing from tone 2 to post-tone 3 (F _2, 25_ = 3.349, *p* = 0.050), with blast-treated rats receiving vehicle freezing more than sham-treated with vehicle (*p* = 0.025, Fisher’s LSD) or blast-treated with HNK (*p* = 0.037). Blast-exposed rats treated with HNK did not differ from sham-treated with vehicle (*p* = 0.961). Error bars in all panels indicate the standard error of the mean (SEM).

Cued fear learning was tested 1 week after (2R,6R)-HNK administration. On day 1 of testing, rats were retrained. All groups trained similarly, exhibiting freezing in response to pairing the tone and the shock (Figure 9C). In the cued fear testing (Figure 9D), blast + vehicle froze more than sham + vehicle (*p* = 0.025) in response to the tone, while blast + (2R,6R)-HNK froze less than blast + vehicle (*p* = 0.037) and no more than sham + vehicle (*p* = 0.961), indicating that exaggerated fear responses had been reversed in blast-exposed female rats treated with (2R,6R)-HNK.

At 32 weeks after blast exposure (2 weeks after [2R,6R]-HNK administration), we retested blast-exposed female rats in a Barnes maze (Supplementary Figure 6). As in early testing at 4 weeks after blast exposure (Supplementary Figure 5), there was no phenotype when comparing sham- and blast-treated rats with saline, and no effect of (2R,6R)-HNK administration.

To test the length of response to (2R,6R)-HNK, we retested animals in the NOR task and cued fear response at 4–5 weeks after drug administration. In NOR testing, the NO from the LTM testing immediately after (2R,6R)-HNK administration was replaced with a different NO. Results are shown in Figure 10A and effect sizes in Supplementary Table 5. Blast + saline did not explore the NO more than the FO [*p* = 0.1245, *g* = 0.8265, 95% CI (−1.595 to 11.60)] while sham + saline [*p* = 0.0051, *g* = 1.172, 95% CI (2.802 to 14.12)] and blast + HNK [*p* = 0.0249, *g* = 1.046, 95% CI (−1.295 to 19.96)] explored the NO more than the FO.

**Figure 10:**
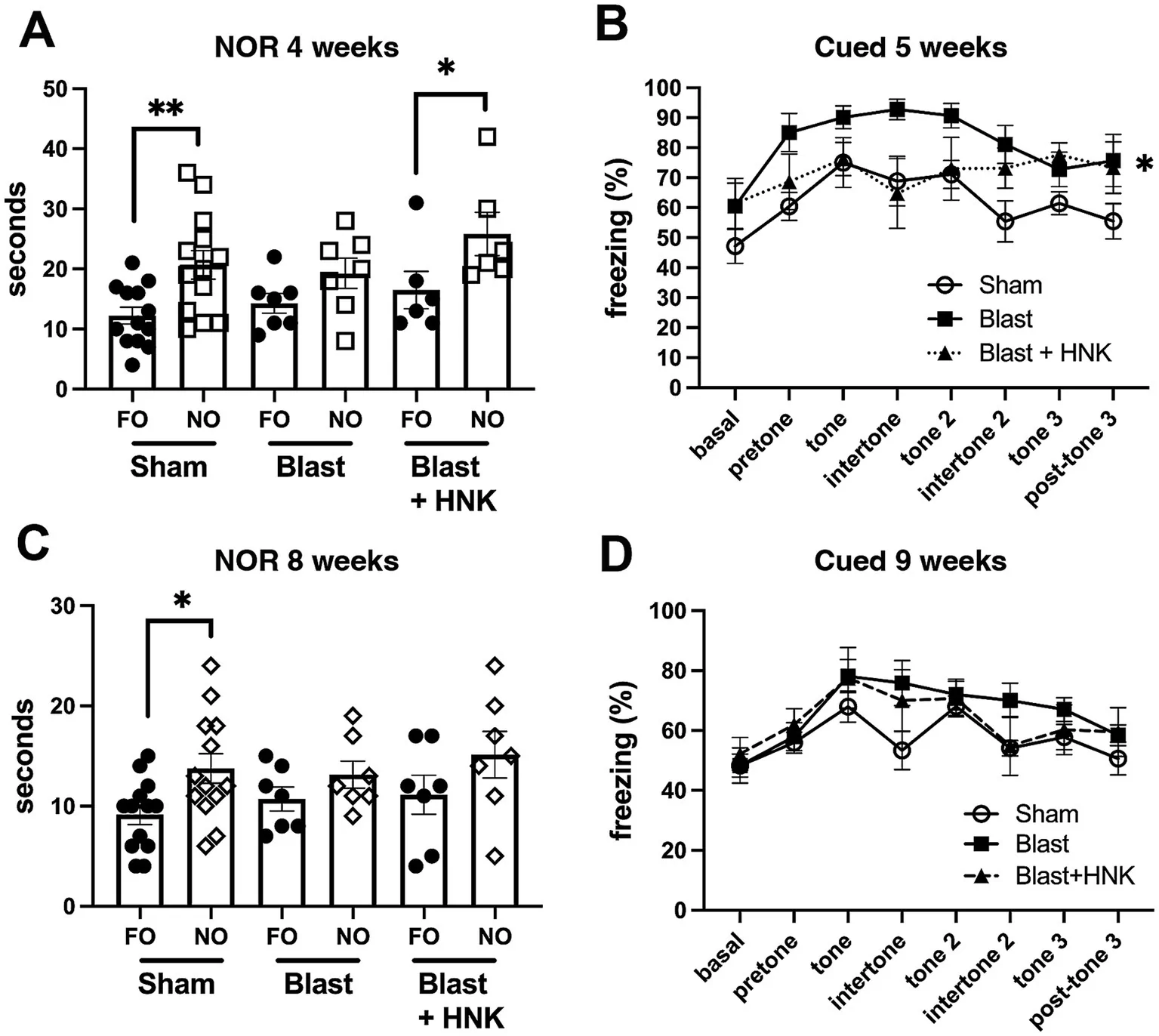
Delayed behavioral testing of female rats treated with (2R,6R)-hydroxynorketamine. Female rats treated with (2R,6R)-HNK (*n* = 14 sham, *n* = 7 blast + saline, *n* = 7 blast + HNK) were retested in NOR and the cued phase of a fear learning paradigm at 4–5 weeks (panels **A** and **B**) or 8–9 weeks (panels **C** and **D**) after administration of a single dose of (2R,6R)-HNK. In NOR testing, the NO from the immediate testing after (2R,6R)-HNK administration (Figure 9) was replaced with a different NO at 4 weeks and replaced again with a new NO at 8 weeks. In the cued fear testing, rats were tested at 5 and 9 weeks after drug administration without retraining. In NOR testing at 4 weeks **(A)**, sham + vehicle and blast + HNK continued to spend more time exploring the NO than the FO (* *p* < 0.05, ** *p* < 0.01, unpaired *t*-tests), while the blast + vehicle explored the objects equally. In cued fear responses at 5 weeks **(B)**, a repeated measures ANOVA of freezing from basal to tone 2 revealed a significant within-subjects effect of freezing for baseline vs. tone (F _2.083_, _43.746_ = 6.974, *p* = 0.002) but no effect of freezing*condition (F _4.166, 43.746_ = 0.693, *p* = 0.606). There were significant overall between-group effects when comparing freezing from basal to tone 2 (F _2, 21_ = 4.939, *p* = 0.017), with blast treated with vehicle freezing more than sham treated with vehicle (*p* = 0.005, Fisher’s LSD) or blast treated with HNK (*p* = 0.049). Blast-exposed treated with HNK did not differ from sham-treated with vehicle (*p* = 0.519). In NOR testing at 8 weeks **(C)**, sham + vehicle spent more time exploring the NO than the FO (** *p* < 0.01, unpaired *t*-tests), while the blast + vehicle and blast + HNK explored the objects equally. In cued fear responses at 9 weeks **(D)** a repeated measures ANOVA revealed significant within-subjects effect with increased freezing across the trials (F _3.752, 82.546_ = 8.397, *p* < 0.001) but no effect of freezing*condition (F _7.504, 82.546_ = 0.919, *p* = 0.501) and there were no significant between-group effects (F _2, 22_ = 1.473, *p* = 0.251). Error bars in all panels indicate the standard error of the mean (SEM).

Cued fear learning was tested by presenting the tone without retraining to the tone + shock. As shown in Figure 10B, as in testing immediately after drug administration, comparing freezing from basal to tone 2, blast treated with vehicle froze more than sham treated with vehicle (*p* = 0.005) or blast treated with HNK (*p* = 0.049), while blast + (2R,6R)-HNK froze no more than sham + vehicle (*p* = 0.519). Thus, responses to (2R,6R)-HNK in both NOR and cued fear learning were sustained at 4–5 weeks after a single drug administration.

To determine whether responses to (2R,6R)-HNK might be maintained longer than 4 weeks, we retested female rats at 8–9 weeks after drug administration. In NOR testing conducted at 8 weeks after drug administration, the FO from the 4-week post-administration testing was replaced with a different object. Results are shown in Figure 10C and effect sizes in Supplementary Table 5. The sham + vehicle continued to explore the NO more the FO [*p* = 0.0156, *g* = 0.9892, 95% CI (0.9575 to 8.273)] while the blast + vehicle [*p* = 0.2025, *g* = 0.6746, 95% CI (−1.496–6.353)] and blast + HNK [*p* = 0.2115, *g* = 0.6605, 95% CI (−2.603 to 10.60)] explored the NO, no more than the FO suggesting that the (2R,6R)-HNK effect had been lost by 8 weeks.

In cued fear learning tested 9 weeks after drug administration, when the rats were presented with the tone without a foot shock, all three groups showed similar freezing profiles (Figure 10D), suggesting that there had been extinction of the cued fear responses in the blast-exposed groups; thus, any (2R,6R)-HNK effect was indeterminate.

### (2R,6R)-hydroxynorketamine

([2R,6R]-HNK) *reverses blast-induced alterations in expression of the metabotropic glutamate receptor mGluR2 but not the serotonin receptor 5-HT2A (5-HT2AR).*

In male rats, we found that mGluR2 is chronically elevated (33) and 5-HT2A is chronically decreased (41). Using tissue collected at the end of the treatment study, we determined whether mGluR2 and 5-HT2AR were chronically altered following blast injury in female rats and whether treatment with (2R,6R)-HNK could reverse these changes. Western blotting for mGluR2 and mGluR3 is shown in Figure 11. As in male rats, mGluR2 was elevated in female rats, and elevations were reversed by (2R,6R)-HNK. mGluR3, which has generally not been found to be affected in male rats (33) was either unchanged or decreased following blast injury depending on the method of normalization (GAPDH vs. total protein) and was not affected by treatment with (2R,6R)-HNK. As in male rats, 5-HT2AR was decreased in blast-injured female rats but levels were not affected by treatment with (2R,6R)-HNK (Figure 12).

**Figure 11:**
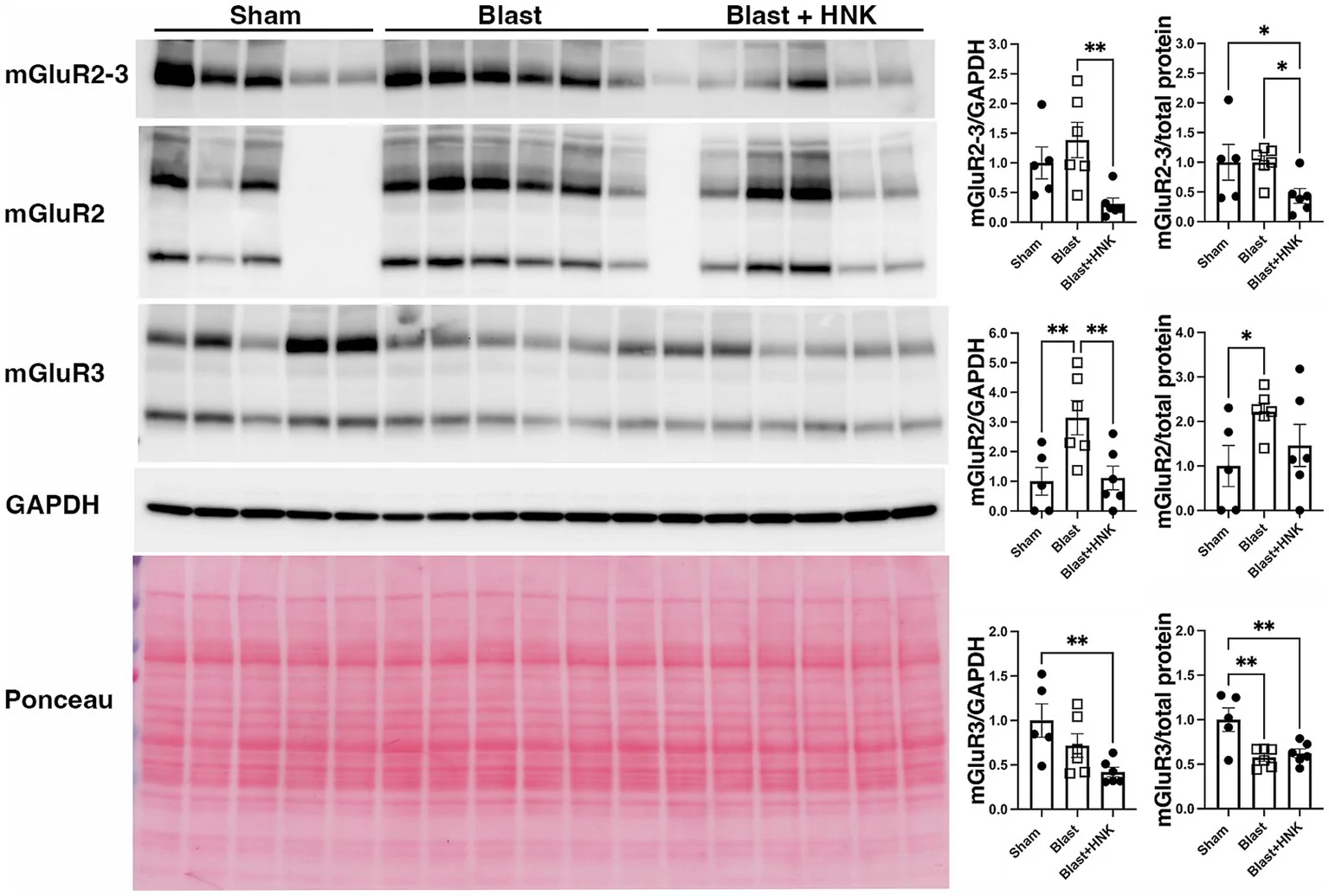
Western blot analysis of mGluR2/3 in female rats treated with (2R,6R)-hydroxynorketamine. mGluR2/3 expression was analyzed in the anterior cortex of control rats (sham, *n* = 5) and blast-exposed rats treated with saline or (2R,6R)-HNK (*n* = 6/group). Panels were probed for mGluR2/3, mGluR2, and glyceraldehyde-3-phosphate dehydrogenase (GAPDH). Targets were sequentially analyzed on the same blot in the order from top to bottom indicated in the Figure. Ponceau S staining of the blot is shown at the bottom. The bar graphs show levels expressed as a ratio to GAPDH or total protein load calculated from the Ponceau S staining. Error bars indicate the SEM in all panels. Asterisks indicate significant differences (Fisher’s LSD after a significant one-way ANOVA, * *p* < 0.05, ** *p* < 0.01).

**Figure 12:**
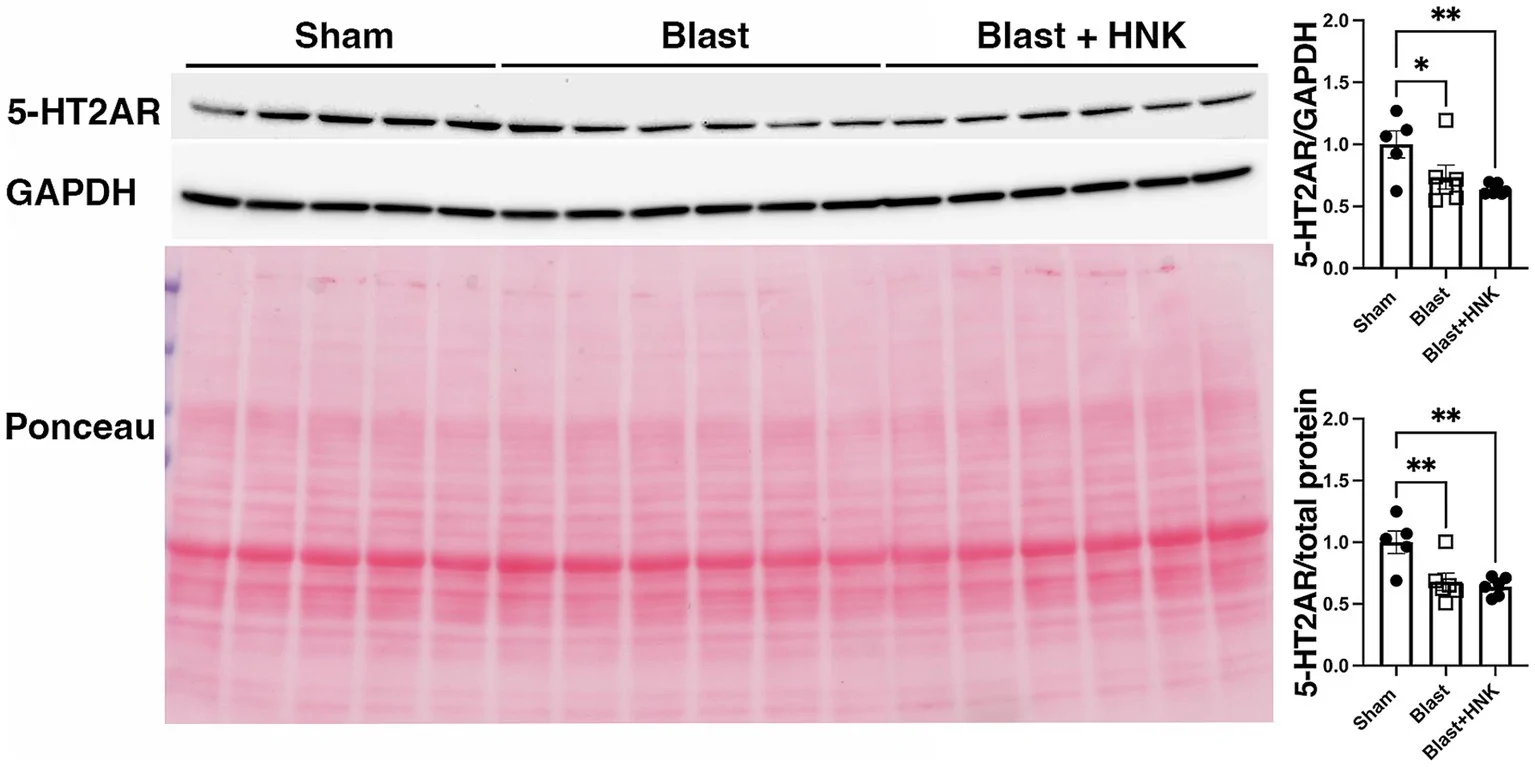
Western blot analysis of 5-HT2AR in female rats treated with (2R,6R)-hydroxynorketamine. 5-HT2AR expression was analyzed in the anterior cortex of control rats (sham, *n* = 5) and blast-exposed rats treated with saline or (2R,6R)-HNK (*n* = 6/group). 5-HT2AR and GAPDH were sequentially analyzed on the same blot in the order from top to bottom indicated in the Figure. Ponceau S staining of the blot is shown at the bottom. The bar graphs show levels of 5-HT2AR expressed as a ratio to GAPDH or total protein load calculated from Ponceau S staining. Error bars indicate the SEM in all panels. Asterisks indicate significant differences (Fisher’s LSD after a significant one-way ANOVA, * *p* < 0.05, ** *p* < 0.01).

## Discussion

A history of TBI is frequent among Veterans seeking care at VA mental health facilities (4). TBI is linked to mental health conditions including anxiety, mood disorders, impaired impulse control, and suicide (42). A striking feature in the recent Veterans from Iraq and Afghanistan has been the association of blast-related mTBI with PTSD (4). How much of this association is driven by blast-related exposure vs. psychological trauma is not clear (4). However, PTSD and depression are the major drivers of worsening functional status in Veterans who suffered concussive blast injuries during service in Afghanistan (11).

PTSD is a mental health disorder that develops after experiencing or witnessing a psychologically traumatizing event (43). Clinical features include re-experiencing phenomena (e.g., flashbacks), as well as hyperarousal symptoms including heightened acoustic startle and avoidance of places or events that serve as reminders of the psychological trauma. Changes in mood and cognition are associated features but not specific to PTSD.

Fear learning in animals is regarded as a critical model for human PTSD (44). Animals exposed to foot shocks in fear-learning paradigms, other stressors, or predator threats develop hyperarousal, avoidance behaviors, and difficulty unlearning fear responses, showing deficits in fear extinction. Rats and humans share highly conserved brain circuits that govern fear learning (44). Fear memories are stored in the amygdala, with the hippocampus and infralimbic and prelimbic frontal cortices regulating fear expression. Current biological models of human PTSD mirror the neurobiology of fear learning in animals, postulating key roles for the prefrontal cortex, amygdala, and hippocampus (45, 46) and suggesting that inadequate inhibition of the amygdala by the prefrontal cortex and hippocampus leads to heightened responses to psychological threats. Functional neuroimaging in humans is consistent with such models, suggesting that in PTSD there is heightened amygdala activity with decreased hippocampal and orbital frontal activity (45).

A hyperactive amygdala in blast-exposed rats is suggested by heightened cued fear responses (8–10). Blast-exposed rats also show heightened responses to a predator scent (47). Thus, blast-exposed rats serve as a model of PTSD-related traits, although these traits are induced by a physical trauma rather than a psychological stressor. We have referred to the condition that these animals model as blast-induced PTSD (48) in the belief that it models a condition different in its pathophysiology from PTSD induced by a psychological stressor. Other components of the blast-induced phenotype, including anxiety (EZM) and impaired cognition (NOR), model associated features of human PTSD but are not specific to the disorder.

Gender differences in response to TBI are well recognized Women exhibit more somatic symptoms and have worse outcomes than men in psychiatric and neurobehavioral measures after TBI (6, 7). They also recover more slowly. Women service members now face essentially the same risks for TBI during military service as male service members. Women service members are also at higher risk for TBI from intimate partner violence, which itself is a risk factor for adverse physical and mental health outcomes (2). Understanding sex-specific responses to TBI and potential treatment is therefore a high priority.

Here we studied 10-week-old female rats exposed to repetitive 75.1 kPa blast overpressure exposures delivered once per day for 3 consecutive days using a blast tube at the James J. Peters VA Medical Center. The blast overpressure wave for these studies was chosen to mimic as closely as possible the blast exposures of our previous studies, which used a shock tube at the Naval Medical Research Command (37). The three-exposure paradigm, given one each day for 3 consecutive days, was chosen to mimic the multiple blast exposures frequently experienced by service members in modern military environments (49, 50).

We tested female rats within 1–4 weeks after blast exposure and again between 28 and 29 weeks after exposure in tasks previously found to be informative in male rats (8–10). We also examined rats in a Barnes maze, which has not been previously studied in this model. Female rats developed a chronic behavioral profile similar to that previously observed in male rats (8–10) exhibiting anxiety in EZM, altered cognition in NOR, and exaggerated cued fear responses. Like males, females exhibited normal contextual fear responses, which have been observed in multiple studies in male rats (8, 9). Females exhibited normal learning in a Barnes maze. Together, these data indicate that chronic blast exposure produces a persistent neurobehavioral phenotype in female rats.

Male rats in this model have been previously used to study treatment with two mGluR2/3 antagonists (33, 51), photobiomodulation (52), boldine (53) and (2R,6R)-HNK (34). (2R,6R)-HNK showed sustained reversal of PTSD-related traits in multiple cohorts of male rats (34). Here, we show that (2R,6R)-HNK is also effective in female rats, with a single dose producing sustained reversal of deficits in NOR and exaggerated cued fear learning for 4 weeks.

Interestingly, the pharmacokinetics of (2R,6R)-HNK differs in male and female mice. After a single dose of (2R,6R)-HNK, female mice show an ≈ 25% higher plasma C_max_ with an ≈ 30–40% increase in the area under the curve (AUC) (25, 27, 54). In this study, we chose to dose female rats with the same 20 mg/kg dose previously administered to male rats (34). If the same sex differences in pharmacokinetics found in mice apply to rats, female rats in this study may have been administered a higher effective dose of (2R,6R)-HNK. Pharmacokinetic studies in male and female rats will be needed to resolve this question. However, whatever the pharmacokinetic differences in male and female rats, these studies provide support for the potential benefit of (2R,6R)-HNK to treat cognitive and mental health disorders following blast injury in females. Future studies using more extended dosing regimens will be needed to determine whether repeated dosing of (2R,6R)-HNK can rescue the behavioral phenotype in males and females for prolonged periods of time.

Mechanistically, it remains unclear why (2R,6R)-HNK might rescue blast-induced behavioral effects. (2R,6R)-HNK exerts rapid and sustained behavioral and cellular effects through mechanisms that are different from ketamine (29, 31, 55). Studies in mice with knockouts of the metabotropic mGluR2 and mGluR3 receptors suggest that (2R,6R)-HNK antidepressant actions are mediated through an mGluR2-dependent mechanism (32). Our initial interest in (2R,6R)-HNK came from observations that the metabotropic glutamate receptor mGluR2 was elevated after blast exposure (33) and that mGluR2/3 receptor antagonists prevented or reversed blast-induced behavioral deficits (33, 51). The finding in this study that (2R,6R)-HNK reversed mGluR2 elevations further supports mGluR2-related mechanisms in (2R,6R)-HNK’s actions. There are sexually dimorphic aspects to glutamatergic neurotransmission (56). Higher mGluR2/3 expression has been reported in the female rat hippocampus and higher mGluR3 expression in the human female cortex (56). What, if anything, this means for (2R,6R)-HNK responses in females vs. males is unclear. Further studies will be needed in males and females to assess how (2R,6R)-HNK affects mGluR2 levels and signaling.

Besides chronic mGluR2 elevation in male rats (33), serotonin receptor 5-HT2A is chronically decreased (41). Both mGluR2 and 5-HT2AR and their G-protein-coupled signaling pathways have been implicated in amygdala-dependent fear processing (57, 58) and hippocampal memory consolidation (59, 60). mGluR2 and 5-HT2AR affect related signaling pathways, having functional as well as reported physical interactions, suggesting that the two receptors act as higher-order complexes (61–63) although the reality of the physical interactions has been questioned (64). Crosstalk between 5-HT2AR and mGluR2 affects responses to antipsychotics and hallucinogens (61, 63). Chronic treatment with the mGluR2/3 antagonist LY341495 also decreases the head twitch response to hallucinogens in wild-type mice (65), and downstream signaling from 5-HT2AR through an HDAC2 histone modification affects mGluR2 gene expression (61, 66). As in male rats, 5-HT2AR was decreased in blast-injured female rats but levels were not affected by treatment with (2R,6R)-HNK. These interactions warrant further study but suggest that reversing 5-HT2AR levels is not required for (2R,6R)-HNK’s therapeutic action.

Several limitations of these studies should be mentioned. These studies examined only one cohort of blast-exposed female rats. Other studies have observed significant differences between male and female mice or rats in their response to blast exposure. For example, Baskin et al. (20) observed that while blast-exposed female and male mice both exhibited acute locomotor and anxiety-like deficits in an open field assay, at 1 month after blast exposure the phenotype only persisted in male mice. Studies have also suggested that female rats may be more resistant than males to acute vascular injury (15, 16) or stressors (23, 24) after blast exposure. Examination of additional cohorts of female rats after blast exposure will be needed to better determine the time course over which the behavioral phenotype develops in females compared to males in this model.

It should also be noted that the three-blast exposure paradigm required repeated anesthesia. Because anesthesia can influence stress responsivity, cognition, and neurochemical signaling, repeated exposure may have contributed to the phenotype. Another methodological limitation is that behavioral testing was performed on the same animals over extended time periods. Repeated testing carries the potential for carryover effects in terms of habituation or learning effects on the later behavioral outcomes. The appearance of an anxiety-like behavior late, as it could affect exploratory drive or emotional reactivity, might have influenced performance in NOR or fear learning. A limitation of the (2R,6R)-HNK studies is that they did not include a sham group treated with (2R,6R)-HNK. Future studies including this group will be needed to determine how (2R,6R)-HNK affects non-blast-exposed Long-Evans rats.

More studies will be needed to determine the effect of blast exposure in female rats on mGluR3, which was either unchanged or decreased following blast injury depending on the method of normalization (GAPDH vs. total protein). Biochemical assessments were also limited to Western blotting for mGluR2/3 and 5-HT2AR. Pathophysiologically, the behavioral phenotype in males is associated with a variety of other vascular, inflammatory, and neurochemical changes along with altered tau processing (67, 68). Studies will be needed to assess whether similar changes are found in females. In this study, data on the estrous cycle were only collected at the time of blast exposure. Future studies will be needed to understand the long-term effects of blast exposure on the estrous cycle and how this affects the chronic behavioral phenotype.

## Conclusion

Male rats subjected to repetitive low-level blast exposure develop chronic cognitive and PTSD-related traits that appear in a delayed manner and remain present more than 1 year after exposure (10). Here, we found that female rats developed a similar set of neurobehavioral alterations that remained present for more than 8 months after blast exposure. These animals thus serve as a model to study the chronic neurobehavioral syndromes that often affect Veterans after blast exposure in males and females (4). As previously seen in male rats (34), a single dose of (2R,6R)-HNK, even when administered after the traits were established, exerted sustained behavioral benifits that persisted at 4–5 weeks without additional doses. Together, these findings support (2R, 6R)-HNK as a promising candidate therapy for blast-related neurobehavioral sequelae.

## Data Availability

The raw data supporting the conclusions of this article will be made available by the authors, without undue reservation.

## Author disclaimer

The views expressed in this study are those of the authors and do not necessarily reflect the official policy or position of the United States Department of Veterans Affairs. Some of the authors are employees of the U. S. Government. This study was prepared as part of their official duties. Title 17 U. S. C. § 105 provides that ‘Copyright protection under this title is not available for any work of the United States Government.’ Title 17 U. S. C. § 101 defines a U. S. Government work as a work prepared by an employee of the U. S. Government as part of that person’s official duties.
