# Supplementary material for: Chronic post-traumatic stress disorder-related behavioral traits develop in female rats exposed to repetitive low-level blast injury and are reversible with (2R,6R)-hydroxynorketamine
Source: Front Neurol. 2026 Oct 7;17:1830745. doi: 10.3389/fneur.2026.1830745 (PMC13683013; doi:10.3389/fneur.2026.1830745)
Supplement: Supplementary file 1 [file Data_Sheet_1.pdf]

Supplemental Figure 1

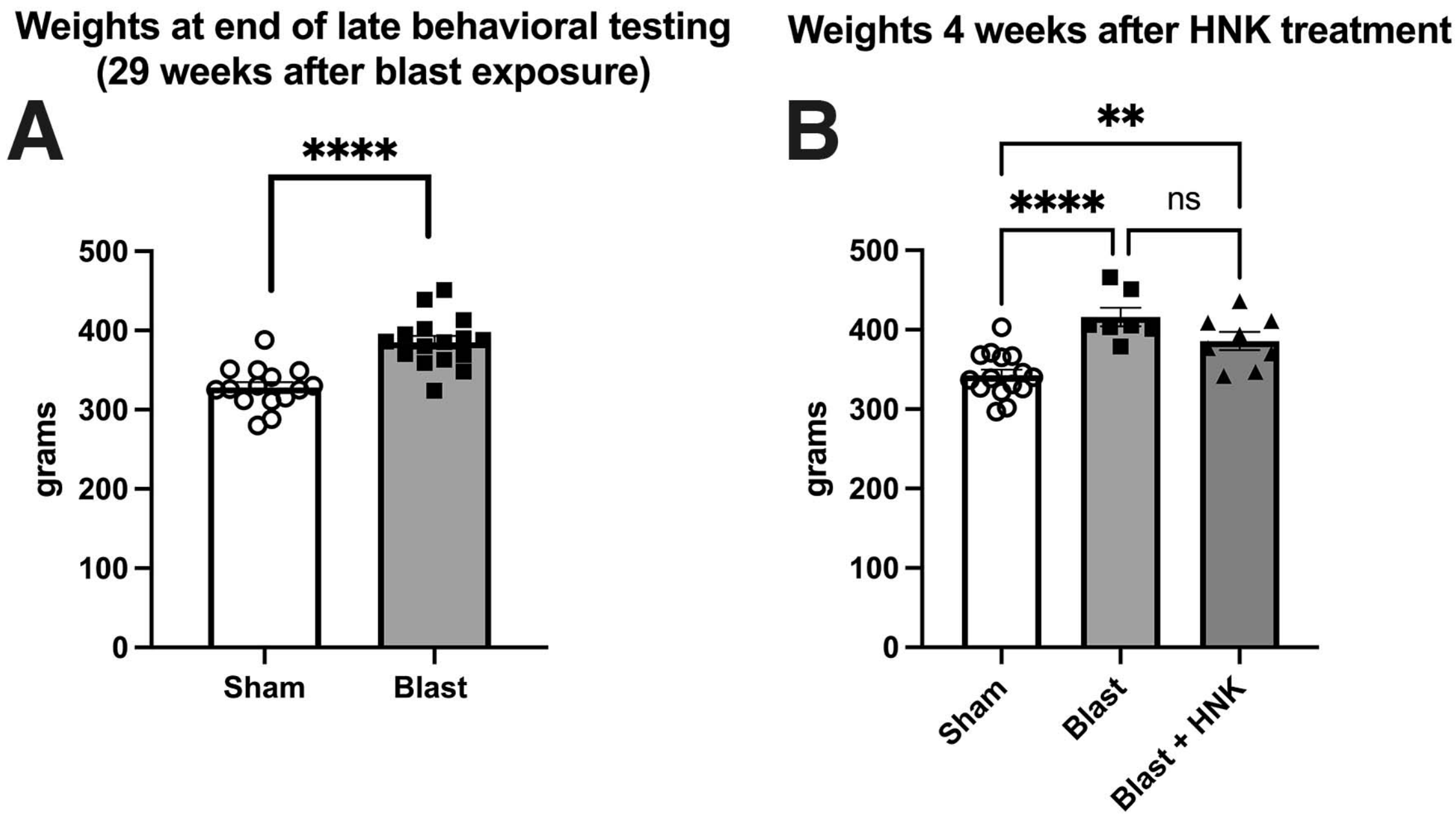

Supplemental Figure 1. Shown in panel **A** are weights at the end of late behavioral testing for blast-exposed (n = 16) and control (n = 15) female rats. Blast exposed female rats weighed ≈ 17% more than controls ($p < 0.0001$, unpaired *t*-test; Hedges' $g = 1.889$, $R^2 = 0.5010$, 95% CI [35.43 to 78.69]). Weights at the end of four weeks of (2R,6R)-HNK treatment are shown in panel **B** (n = 15 sham, n = 7 blast + saline, n =8 blast + HNK). A one-way ANOVA revealed significant group differences ($F_{2, 27} = 15.46$, $p < 0.0001$) with controls + saline weighing less than blast + saline ($p < 0.0001$, Fisher's LSD) or blast + HNK ($p = 0.0029$) while blast + saline compared to blast + HNK did not differ ($p = 0.0629$). Error bars in all panels indicate the standard error of the mean (SEM). Asterisks indicate values significantly different (** $p < 0.01$, **** $p < 0.0001$, ns not significant).

Supplemental Figure 2

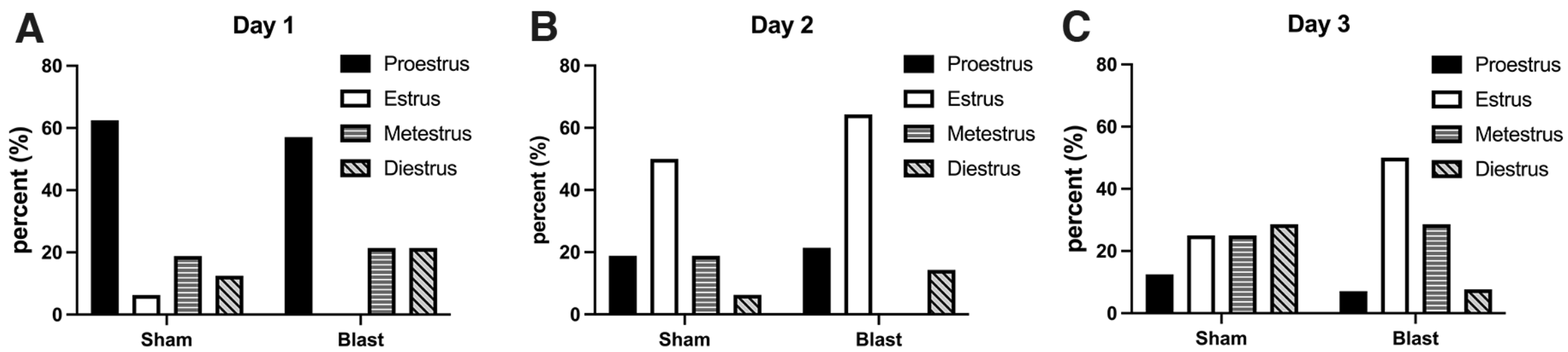

Supplemental Figure 2. Estrous cycle in female rats before and during blast exposure sequence. The graphs show the stage of the estrous cycle recorded before blast exposure on day 1 (**A**), day 2 (**B**) and day 3 (**C**) in blast-exposed (n = 16) and control (n = 16) female rats. Data is presented as the percent (%) in each phase of the cycle. On day 1 (before blast exposure of either group) there were no differences in the relative frequencies of the stage of the estrous cycle between the groups ($p = 0.9931$, Fisher's exact test). Relative frequencies also did not differ when examined prior to the blast exposures on day 2 ($p = 0.4933$) or day 3 ($p = 0.4811$).

Supplemental Figure 3

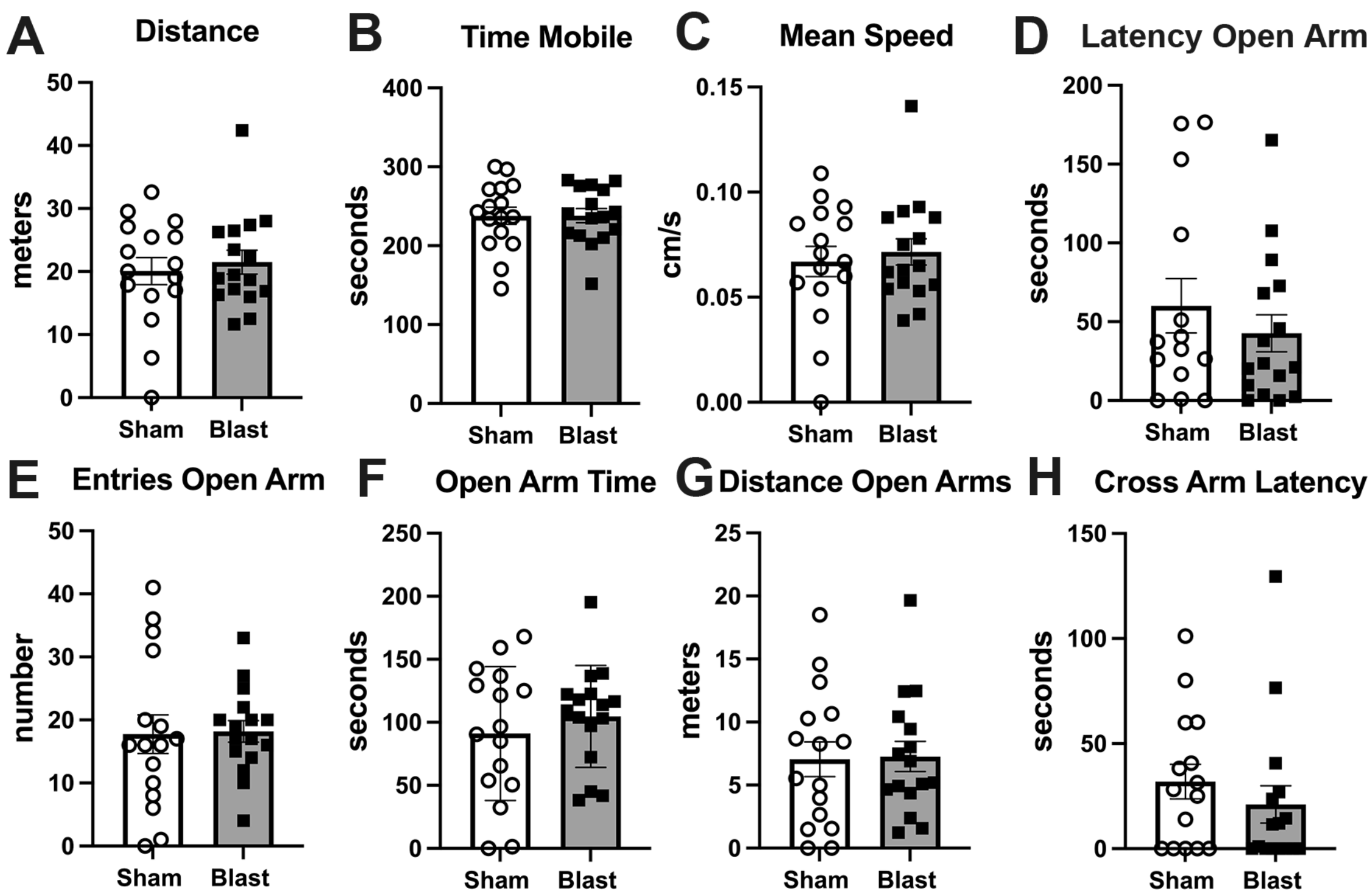

Supplemental Figure 3. Elevated zero maze **(**EZM) testing of females within 2 weeks after blast exposure. Blast-exposed (n = 16) and control (n = 16) female rats were tested in an EMZ. The plots show the distance traveled **(A)**, time in motion **(B)**, mean speed **(C)**, latency to enter an open arm **(D)**, open arm entries **(E)**, time spent in the open arms **(F),** distance traveled in an open arm **(G)** and the latency to cross into a second open arm **(H)**. There were no statistically significant differences between the groups in any measure (unpaired *t*-tests).

Supplemental Figure 4

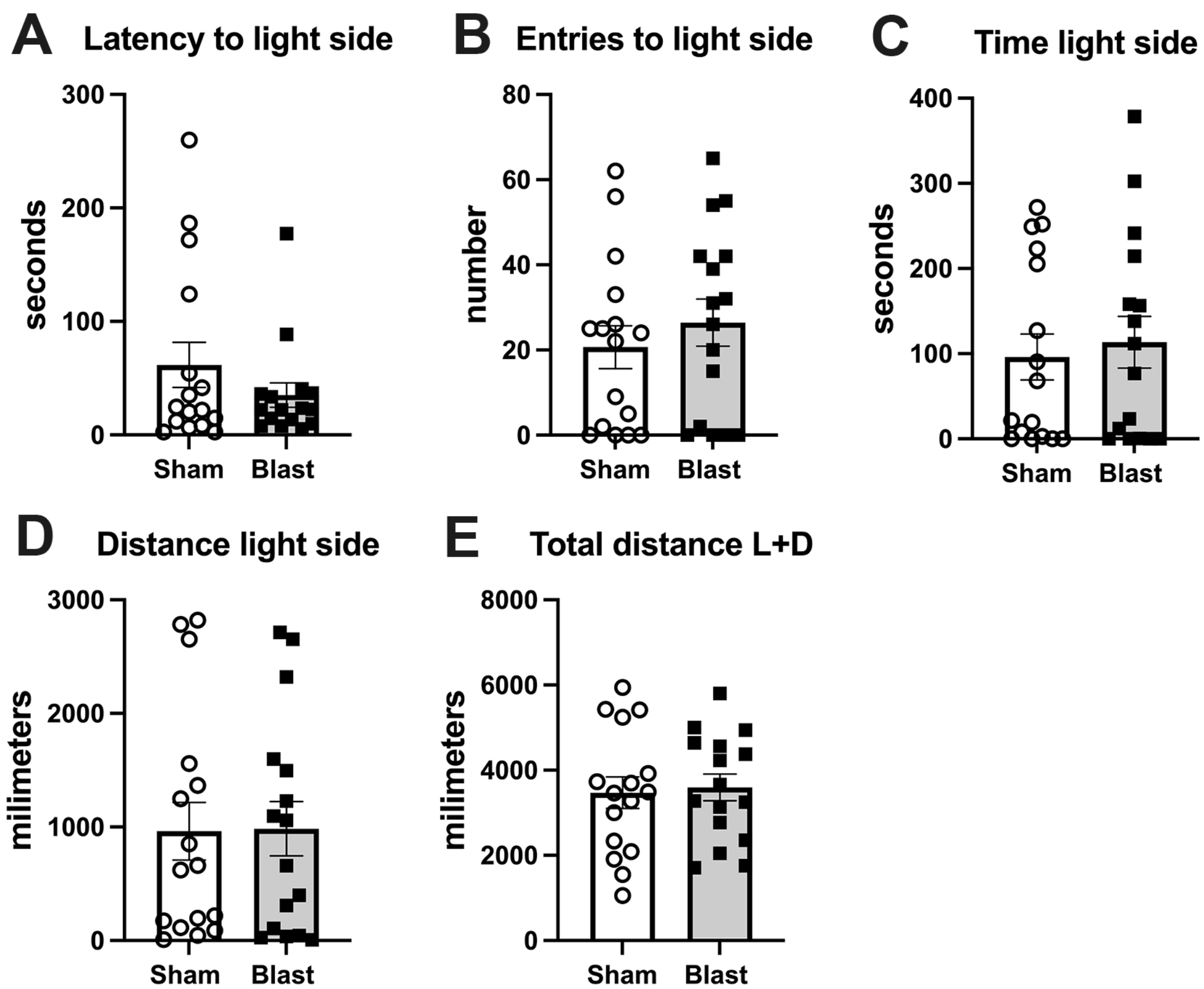

Supplemental Figure 4. Light dark (LD) emergence testing of females within 1 week after blast exposure. Blast-exposed (n = 16) and control (n = 16) female rats were tested in a LD emergence task. The plots show the latency to enter the light side **(A)**, entries into the light side **(B)**, time on the light side **(C),** distance moved on the light side **(D)** and total distance moved light plus dark **(E)**. Error bars indicate the standard error of the mean (SEM). There were no statistically significant differences between the groups in any measure (unpaired *t*-tests).

Supplemental Figure 5

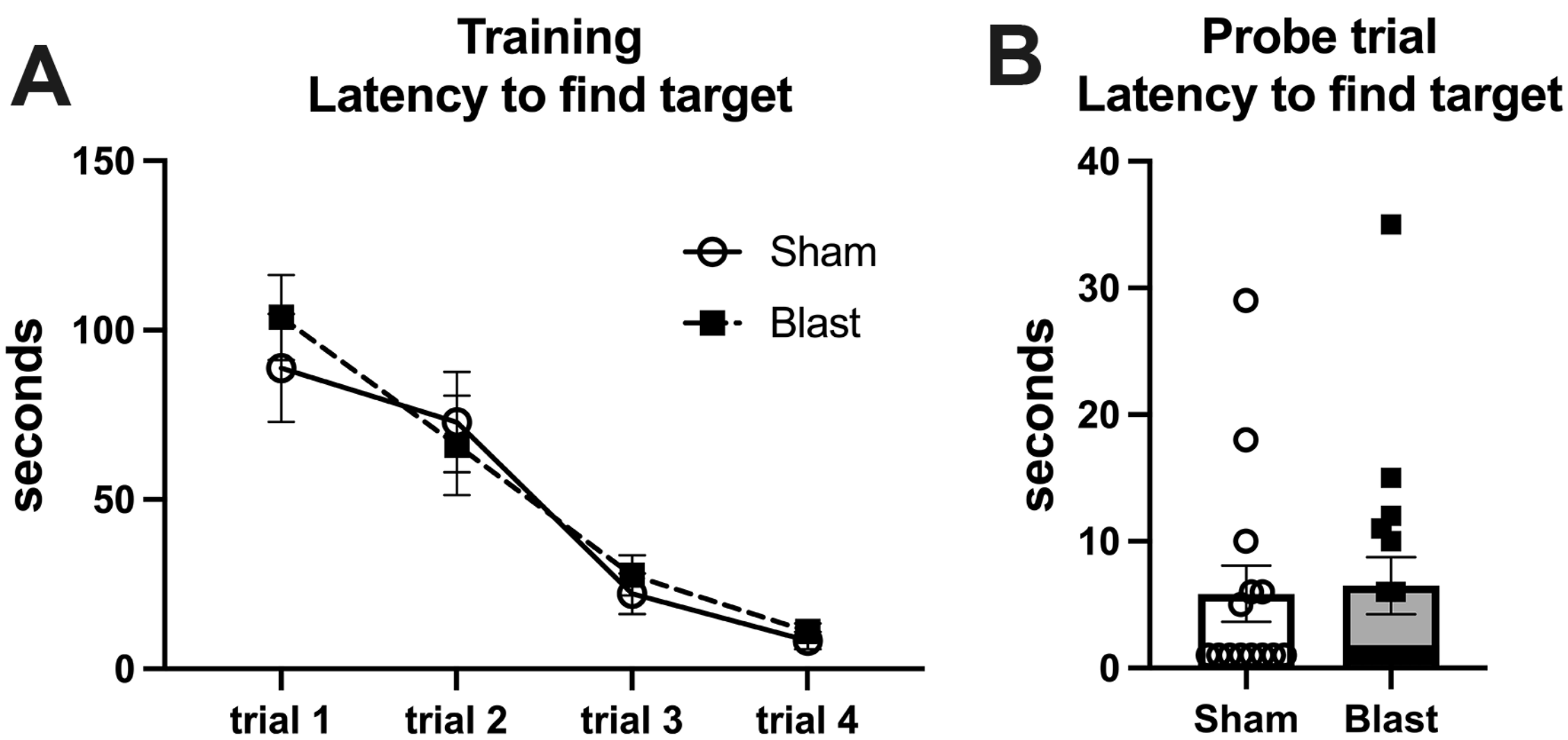

Supplemental Figure 5. Testing of blast-exposed female rats in a Barnes maze at 4 weeks after exposure. Blast-exposed (n = 16) and sham-exposed (n = 16) female rats were tested in a Barnes maze. The latency to find the target is shown for the training **(A)** and probe trial **(B)**. Both groups responded with decreasing latency to enter the target quadrant ($F_{1.778, 51.572}$ = 24.885, p = < 0.001) without an interaction effect ($F_{1.778, 51.572}$ = 0.312, p = 0.707) and no overall differences between groups ($F_{1, 29}$ = 0.425, p = 0.519). In the probe trial, the latency to find the target did not differ between groups (p = 0.8411, unpaired *t*-test).

Supplemental Figure 6

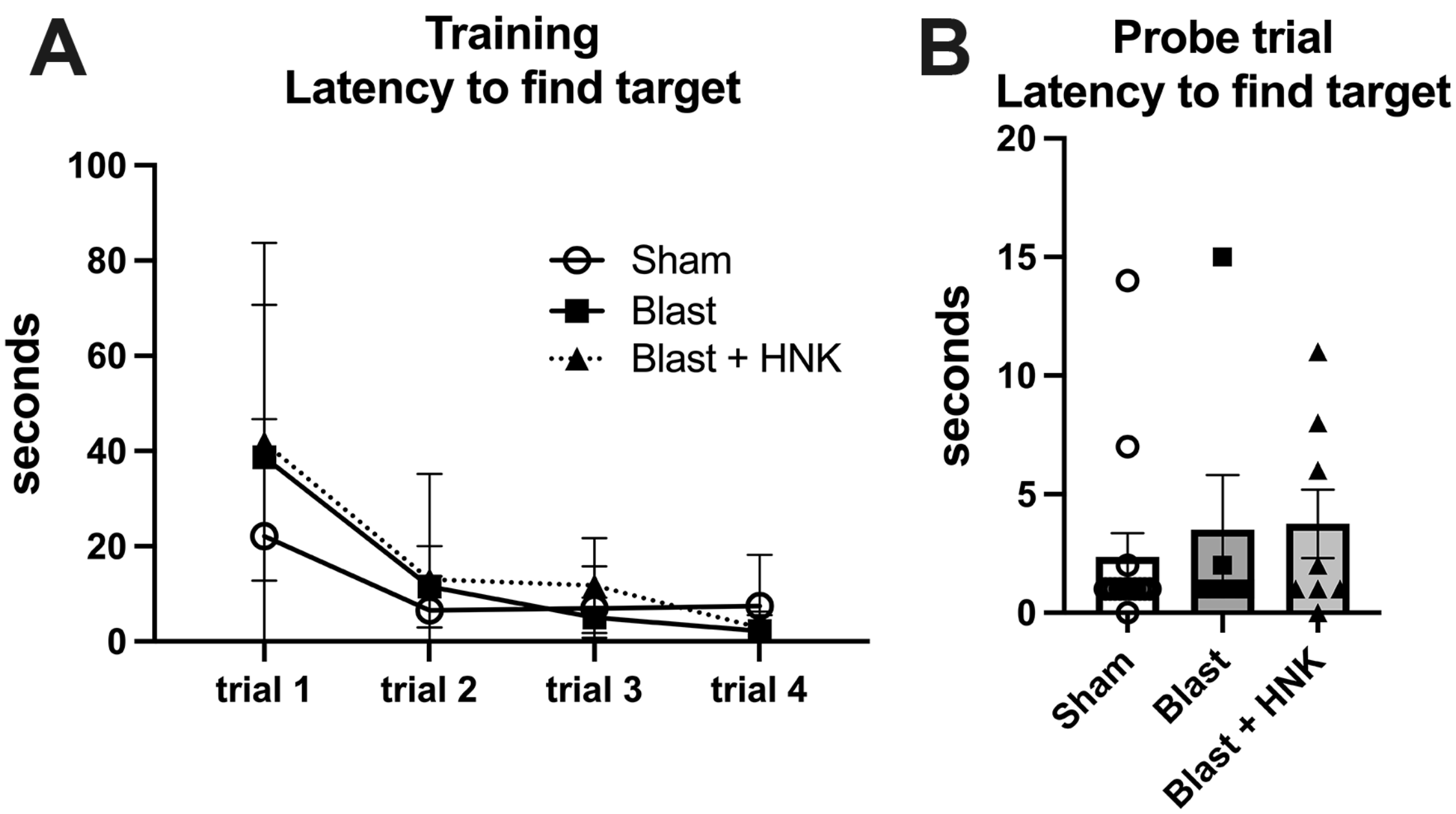

Supplemental Figure 6. Testing of blast-exposed females treated with (2R,6R)-hydroxynorketamine in a Barnes maze. Sham and blast-exposed female rats were treated with saline (n = 15 sham, n = 7 blast) or blast were treated with (2R,6R)-HNK (n = 8). The latency to find the target is shown for the training **(A)** and probe trial **(B)**. All groups responded with decreasing latency to enter the target quadrant ($F_{1.685, 42.113} = 17.252$, $p = < 0.001$) without an interaction effect ($F_{3.369, 42.113} = 1.160$, $p = 0.339$) and no overall differences between groups ($F_{2, 25} = 1.143$, p = 0.335). In the probe trial, the latency to find the target did not differ between groups (one way ANOVA, $F_{2, 25} = 0.323$, $p = 0.727$). Error bars in all panels indicate the standard error of the mean (SEM).

Supplemental Table 1

Progressive staining steps used for vaginal smears to determine the stage of the estrous cycle.

Step	Solution	Time per change	Repeat step
1	95% ethanol	5 min.	1
2	tap water	10 quick dips	1
3	Gill's Hematoxylin	2 min.	0
4	tap water	10 quick dips	1
5	Scott's tap water substitute	1 min.	0
6	tap water	10 quick dips	1
7	95% ethanol	10 quick dips	1
8	Orange G6	1 min.	0
9	95% ethanol	10 quick dips	1
10	eosin-azure 50	10 min.	0
11	95% ethanol	20 quick dips	2
12	100% ethanol	10 quick dips	2
13	xylene	10 quick dips	2
14	Mounting		

Supplemental Table 2

Effect sizes for novel object recognition (NOR) testing shown Figure 4 in the text.

**NOR Figure 4**	*t*-test *p* value	Hedges' *g*	R^2 (eta squared)	95% confidence interval
**Training**				
Sham	0.7713	0.1011	0.002863	-2.236 to 2.986
Blast	0.8226	0.07798	0.001782	-2.011 to 2.511
**STM**				
Sham	0.0002	1.490	0.3967	4.245 to 11.88
Blast	0.0954	0.5934	0.09266	-0.5046 to 5.880
**LTM**				
Sham	0.0018	1.179	0.3709	4.023 to 16.60
Blast	0.7205	0.1245	0.004764	-3.801 to 5.426
**DI**				
STM	0.0541	-0.6909	0.1224	-0.3913 to 0.003960
LTM	0.0498	-0.7065	0.1473	-0.4651 to 0.001394

Supplemental Table 3

Effect sizes for elevated zero maze (EZM) testing shown Figure 6 in the text.

**EZM**<br>**Figure 6**	*t*-test<br>*p* value	Hedges' *g*	R^2 (eta squared)	95% confidence interval
**Day1**				
Distance	0.1922	-0.4748	0.05996	-8.322 to 1.750
Mean speed	0.3088	-0.3682	0.03695	-0.02699 to 0.008852
Time mobile	0.7669	-0.1064	0.003190	-25.41 to 18.93
Latency open arm	0.0202	0.8604	0.1724	3.912 to 42.68
Entries open arm	0.0412	-0.7745	0.1455	-12.43 to -0.2741
Open arm time	0.0412	-0.7480	0.1360	-67.27 to -1.476
Cross arm latency	0.0178	0.8968	0.1847	6.747 to 65.54
**Day 2**				
Distance	0.2771	-0.3996	0.04391	-10.55 to 3.146
Mean speed	0.1318	-0.5709	0.08518	-0.03846 to 0.005321
Time mobile	0.6827	-0.1445	0.005844	-36.43 to 24.19
Latency open arm	0.0017	1.210	0.2917	13.91 to 54.24
Entries open arm	0.0144	-0.9114	0.1895	-17.10 to -2.055
Open arm time	0.0034	-1.161	0.2768	-57.25 to -12.64
Cross arm latency	0.0122	0.9362	0.1979	15.10 to 113.2

Supplemental Table 4

Effect sizes for novel object recognition (NOR) testing shown Figure 7 in the text.

**NOR Figure 7**	*t*-test *p* value	Hedges' *g*	R^2 (eta squared)	95% confidence interval
**Training**				
Sham	0.9717	0.01270	4.560e-005	-3.755 to 3.888
Blast	0.8100	0.08359	0.001957	-2.783 to 3.533
**STM**				
Sham	0.0057	1.063	0.2422	2.627 to 14.04
Blast	0.8882	-0.04887	0.0006697	-5.776 to 5.026
**LTM**				
Sham	0.0002	1.502	0.3895	9.071 to 26.13
Blast	0.2113	0.4402	0.05158	-2.358 to 10.23
**DI**				
STM	0.0004	-1.424	0.3634	-0.4765 to -0.1536
LTM	0.0002	-1.510	0.3921	-0.4673 to -0.1633

Supplemental Table 5

Effect sizes for novel object recognition (NOR) testing shown Figures 9 and 10 in the text.

**NOR**	*t*-test *p* value	Hedges' *g*	R^2 (eta squared)	95% confidence interval
**Figure 9A-B**				
**Training**				
Sham	0.6282	-0.1739	0.008488	-5.875 to 3.609
Blast	0.6372	0.2421	0.01914	-4.504 to 7.075
Blast + HNK	0.9472	0.03186	0.0003243	-7.707 to 8.207
**Memory 24h**				
Sham	0.0022	1.245	0.3070	3.013 to 12.27
Blast	0.6778	0.2130	0.01488	-4.118 to 6.118
Blast + HNK	0.0119	0.9259	0.2151	-0.8082 to 17.81
**Figure 10A/C**				
**LTM 4 weeks**				
Sham	0.0051	1.172	0.2840	2.802 to 14.12
Blast	0.1245	0.8265	0.1852	-1.595 to 11.60
Blast + HNK	0.0249	1.046	0.2827	-1.295 to 19.96
**LTM 8 weeks**				
Sham	0.0156	0.9892	0.2203	0.9575 to 8.273
Blast	0.2025	0.6746	0.1315	-1.496 to 6.353
Blast + HNK	0.2115	0.6605	0.1268	-2.603 to 10.60
